# Performance and operational feasibility of two diagnostic tests for cryptosporidiosis in children (CRYPTO-POC): a clinical, prospective, diagnostic accuracy study

**DOI:** 10.1016/S1473-3099(20)30556-9

**Published:** 2021-05

**Authors:** Øystein H Johansen, Alemseged Abdissa, Mike Zangenberg, Zeleke Mekonnen, Beza Eshetu, Ola Bjørang, Yonas Alemu, Bizuwarek Sharew, Nina Langeland, Lucy J Robertson, Kurt Hanevik

**Affiliations:** a Department of Clinical Science, University of Bergen, Bergen, Norway; b Department of Microbiology, Vestfold Hospital Trust, Tønsberg, Norway; c School of Medical Laboratory Sciences, Jimma University, Jimma, Ethiopia; d Department of Paediatrics and Child Health, Jimma Institute of Health, Jimma University, Jimma, Ethiopia; e Department of Immunology and Microbiology, Centre for Medical Parasitology, University of Copenhagen, Copenhagen, Denmark; f Department of Clinical Microbiology, Copenhagen University Hospital (Rigshospitalet), Copenhagen, Denmark; g Norwegian National Advisory Unit on Tropical Infectious Diseases, Department of Medicine, Haukeland University Hospital, Bergen, Norway; h Parasitology, Department of Paraclinical Sciences, Faculty of Veterinary Medicine, Norwegian University of Life Sciences, Oslo, Norway

## Abstract

**Background:**

Cryptosporidiosis is a common cause of diarrhoea in young children (aged younger than 24 months) in low-resource settings but is currently challenging to diagnose. Light-emitting diode fluorescence microscopy with auramine-phenol staining (LED-AP), recommended for tuberculosis testing, can also detect *Cryptosporidium* species. A lateral-flow test not requiring refrigerator storage (by contrast with most immunochromatographic lateral-flow assays) has also recently been developed for *Cryptosporidium* spp detection. We aimed to evaluate the diagnostic accuracy and operational feasibility of LED-AP and the lateral-flow test strip for cryptosporidiosis in children.

**Methods:**

We did a prospective diagnostic accuracy study in two health-care facilities in Ethiopia, in a consecutive series of children younger than 5 years of age with diarrhoea (three or more loose stools within the previous 24 h) or dysentery (at least one loose stool with stains of blood within the previous 24 h). Stool samples were tested for *Cryptosporidium* spp by LED-AP and the lateral-flow test strip; accuracy of each test was estimated by independent and blind comparison with a composite reference standard comprising quantitative immunofluorescent antibody test (qIFAT), ELISA, and quantitative PCR (qPCR). Quantitative cutoff values for diarrhoea-associated infection were established in an embedded case-control substudy, with cases of cryptosporidiosis coming from the 15 districts in and around Jimma and the eight districts surrounding Serbo, and community controls without diarrhoea in the previous 48 h recruited by weekly frequency matching by geographical district of the household, age group, and enrolment week.

**Findings:**

Stool samples from 912 children with diarrhoea or dysentery and 706 controls from the case-control substudy were tested between Dec 22, 2016, and July 6, 2018. Estimated reference-standard cutoff values for cryptosporidiosis positivity were 2·3 × 10^5^ DNA copies per g of wet stool for qPCR, and 725 oocysts per g for qIFAT. LED-AP had a sensitivity for cryptosporidiosis of 88% (95% CI 79–94; 66 of 75 samples) and a specificity of 99% (98–99; 717 of 726 samples); the lateral-flow test strip had a sensitivity of 89% (79–94; 63 of 71 samples) and a specificity of 99% (97–99; 626 of 635 samples).

**Interpretation:**

LED-AP has high sensitivity and specificity for cryptosporidiosis and should be considered as a dual-use technology that can be easily integrated with existing laboratory infrastructures in low-resource settings. The lateral-flow test strip has similar sensitivity and specificity and provides an alternative that does not require microscopy, although purchase cost of the test strip is unknown as it is not yet available on the market.

**Funding:**

Norwegian Research Council GLOBVAC fund, The Bill & Melinda Gates Foundation, Norwegian Society for Medical Microbiology, University of Bergen, and Vestfold Hospital Trust.

## Introduction

Cryptosporidiosis is the fifth leading cause of diarrhoeal mortality worldwide and caused more than 48 000 deaths and led to a loss of 12·9 million disability-adjusted life-years in 2016, with the highest burden in sub-Saharan Africa.^1^ The disease ranked among the top five causes of diarrhoea in the Global Enteric Multicenter Study in 2007–11,^2^, ^3^ and has probably increased in relative importance since the rotavirus vaccine was rolled out globally.^4^ There is no vaccine against *Cryptosporidium* species and the only approved drug, nitazoxanide, has moderate effect on diarrhoea and parasite clearance; although the drug significantly reduced mortality in one trial, it is not effective in children with HIV.^5^ Increased effort has been put into developing better drugs for cryptosporidiosis,^6^ but a test-and-treat strategy will require a rapid, low-technology, reliable, and affordable diagnostic test that can be used near the point of care, and that is suitable for use in low-resource settings.^7^ Diagnostic accuracy evaluations that are not done in the relevant clinical setting overestimate diagnostic accuracy and field applicability.^8^ We identified only one study from east Africa that evaluated immunochromatographic lateral-flow assays (ICLFs) in children with complicated severe acute malnutrition, reporting moderate sensitivity against PCR results,^9^ and a Turkish study that evaluated modified Ziehl-Neelsen staining microscopy.^10^ The Turkish study concluded, concurring with many previous reports, that modified Ziehl-Neelsen staining microscopy is insufficiently accurate.^5^, ^11^

**Research in context**

**Evidence before this study**

*Cryptosporidium* species are a common cause of watery childhood diarrhoea and an important cause of morbidity and mortality, particularly in sub-Saharan Africa. Current treatment for cryptosporidiosis is suboptimal, despite new drugs being in development, which emphasises the need for efficient and effective diagnostics. Most diagnostic tests in current use are either inaccurate or satisfy few of the commonly recommended criteria for an ideal diagnostic test for resource-limited settings. Targeted treatment strategies should be promoted on the basis of evidence of accuracy and operational performance from studies in representative clinical settings under field conditions.

We did a semisystematic review within PubMed using the search terms “cryptosporid*” AND (“test” OR “microscop*” OR “assay” OR “detect*”) AND (“accuracy” OR “sensitivity” OR “specificity” OR “diagnos*” OR “trial*”) AND (“diarrh*” OR “gastroenteritis” OR “gastrointestinal symptoms”) for articles in all languages, published from database inception to Jan 20, 2020, to identify studies on diagnostic accuracy for *Cryptosporidium* spp infection that included information on diarrhoeal symptoms with a minimum of 50 positive and 50 negative samples. We initially searched for prospective studies in consecutive series of children in low-income or middle-income countries (LMICs), as this is the recommended study design for external validity. Two studies were identified: one study from a middle-income country (Turkey) that reported 40% sensitivity of the traditional modified Ziehl-Neelsen staining microscopy method against ELISA, and one study from east Africa (Kenya and Malawi) that reported moderate sensitivity (43–77%) of three immunochromatographic lateral-flow (ICLF) tests against any detection by PCR—this was, however, a study in children with complicated severe acute malnutrition, and the estimates might not apply to the general paediatric population. Two studies from Bangladesh (one retrospective) reported good accuracy of ICLFs against diarrhoea attributed to *Cryptosporidium* spp by quantitative PCR; however, they used samples from a birth cohort study, with repeated samples, and their findings might not extrapolate to clinical settings.

A retrospective multicentre study from the UK reported 92% sensitivity and 100% specificity of auramine-phenol (AP) staining against PCR and immunofluorescent antibody tests, but we identified no clinical diagnostic accuracy studies on the use of AP for cryptosporidiosis in children in LMICs. Furthermore, we did not find any field studies in LMICs that included data on operational issues, such as comprehensive cost calculations and test turnaround times.

**Added value of this study**

We evaluated the diagnostic accuracy of two simple tests (light-emitting diode fluorescence microscopy with AP staining [LED-AP] and a lateral-flow test strip) for cryptosporidiosis in children in a low-income country, and included cost-per-test calculations and operational assessment. Asymptomatic infections were controlled for by using quantitative reference methods. The sensitivity of LED-AP was consistent with estimates from a study in a high-income country, while maintaining high specificity. The lateral-flow test strip had similar sensitivity and specificity to LED-AP.

**Implications of all the available evidence**

Test-and-treat strategies for diarrhoea that include accurate detection of the aetiological agent will increase the chance of a useful clinical response. LED-AP is a reliable and affordable test for cryptosporidiosis that can be integrated with existing laboratory infrastructure, near the point of care, in LMICs. The lateral-flow test strip could be an alternative when LED-AP is unavailable, but is likely to be more expensive. Laboratories in LMICs that use modified Ziehl-Neelsen staining microscopy for *Cryptosporidium* spp testing could consider switching to LED-AP.

In 2011, WHO recommended a large-scale shift in first-line tuberculosis testing, to light-emitting diode fluorescence microscopy with auramine-phenol (AP) staining (LED-AP).^12^ Available at a reduced price to low-income countries, thousands of LED microscopes have been distributed in Ethiopia and other low-income or middle-income countries (LMICs).^13^ AP microscopy, using traditional halogen-bulb fluorescence microscopes, is well established as a diagnostic test for *Cryptosporidium* spp,^11^, ^14^ but its field performance in high-prevalence settings is largely unknown, with the exception of a small study in HIV-positive adults in India that reported high accuracy.^15^ We found no studies that evaluated AP microscopy against the most accurate reference-standard tests (immunofluorescence antibody test [IFAT] microscopy or quantitative PCR [qPCR]) in children in an LMIC.

We hypothesised that LED-AP would have acceptable diagnostic accuracy (minimum sensitivity 70%, specificity 96%) and operational feasibility for cryptosporidiosis testing in children presenting to health-care facilities in a low-resource setting with high rates of malnutrition. As the most likely next-best alternative in centres without microscopy facilities, we included an ICLF in the evaluation; although ICLFs have variable performance in high-income countries,^16^, ^17^, ^18^ a few have shown promising results in LMICs.^9^, ^19^, ^20^ Most ICLFs require refrigeration; however, a lateral-flow test strip for *Cryptosporidium* spp that can be stored at room temperature has now been developed.^20^ We aimed to evaluate the diagnostic accuracy and operational feasibility of LED-AP and this lateral-flow test strip for cryptosporidiosis.

Few diagnostic accuracy studies and trials distinguish between *Cryptosporidium* spp infection and cryptosporidiosis, despite asymptomatic infections being well described for *Cryptosporidium* spp and other diarrhoeal pathogens. Simply detecting a pathogen will have reduced value when the purpose of testing is to select those patients for whom intervention might be of clinical value. Building on studies where quantitative testing in both cases of cryptosporidiosis and community controls has been applied to discriminate between asymptomatic and symptomatic infection,^2^, ^21^, ^22^ we chose a panel of reference tests that included two different quantitative assays. We embedded a case-control study in the accuracy study to estimate quantitative cutoff values, and to allow estimation of the predictive value of LED-AP and the ICLF test strip for clinical disease in our study population.

## Methods

### Study design and participants

We did a prospective diagnostic accuracy study for cryptosporidiosis in children presenting to health-care facilities with diarrhoea or dysentery, in Jimma Medical Centre, Jimma, and Serbo Health Centre, Serbo, in southwest Ethiopia. Jimma Medical Centre is a tertiary referral hospital located in an urban area; Serbo Health Centre covers a rural area around the smaller town of Serbo, approximately 16 km from Jimma.

Study nurses screened children younger than 5 years of age at the paediatric outpatient departments and all inpatient wards at both centres for eligibility. Children were consecutively enrolled from 0800 h to 1800 h every day at Jimma Medical Centre and from 0800 h to 1700 h in Serbo Health Centre. Children were eligible if they had diarrhoea (three or more loose stools within the previous 24 h) or dysentery (at least one loose stool with stains of blood within the previous 24 h), regardless of whether these were the primary complaints leading them to seek health care. The exclusion criterion was inpatient admission for longer than 24 h before enrolment in the study. Written informed consent was obtained from the children's caregivers and we followed STARD guidelines.^8^

The embedded case-control study included children with diarrhoea from the 15 districts in and around Jimma and the eight districts surrounding Serbo. Community controls without diarrhoea in the previous 48 h were recruited by weekly frequency matching by geographical district of the household, age group, and enrolment week.^23^

Jimma University Institutional Review Board (reference RPGC/610/2016), the Ethiopian National Research Ethics Review Committee (reference JU JURPGD/839/2017), and the Regional Committee for Medical and Health Research Ethics of Western Norway (reference 2016/1096) approved the study.

### Procedures

Study nurses collected demographic and clinical data using standardised case-report forms. Information about treatment and clinical outcome was collected by interview with caregivers and from hospital records. All participants were tested for HIV (appendix 2 p 2), where possible, and were asked to provide a stool sample. Stool was collected using a nappy, single-use bedpan, or potty, and quickly transferred to a screw-cap plastic container. Study nurses lined nappies with plastic film to avoid water absorption from the stool into the absorbent polymer layer of the nappy. Samples were stored at 4°C until further processing.

**Figure 1:**
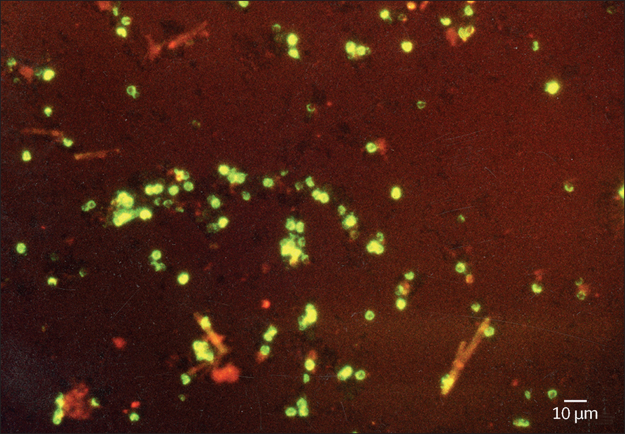
Auramine-phenol stained *Cryptosporidium* spp oocysts (diameter 4–6 μm), displaying characteristic erythrocyte pattern of staining Section of 400 × magnification field (LJR, personal photograph).

All laboratory personnel that performed index and reference testing were fully masked to results of the other tests and to clinical information, including case or control status (ie, diarrhoea or no diarrhoea) of the participants. LED-AP testing was done by medical laboratory technicians trained in the method according to a described standardised operating procedure (appendix 2 p 16) in addition to their routine laboratory duties. AP staining was done on a manually homogenised, air-dried stool smear, without preceding sample concentration. The reagents used were the same as those used for auramine staining of sputum smears for acid-fast bacilli, but with a standard operating procedure optimised for detecting *Cryptosporidium* spp oocysts. AP-stained slides were examined using a PrimoStar iLED fluorescence microscope (Carl Zeiss Microscopy, Jena, Germany) for objects of the same size and morphology as *Cryptosporidium* spp oocysts (figure 1) and were categorised according to average number of oocysts per 200 × magnification field of view: one to nine, ten to 50, or more than 50 (appendix 2 pp 2, 17).

Findings were reported on a standardised results form. Microscopy slides were kept at ambient temperature in a closed, non-transparent box. Once per month, the study investigator (ØHJ) selected ten slides for blinded review and to assess microscope settings and the quality of stains (appendix 2 pp 21–22); result discrepancies did not affect the recorded results of LED-AP testing, but were used as a basis for immediate feedback and on-site retraining for laboratory technicians.

*Cryptosporidium* EZ VUE lateral-flow test strips (TECHLAB, Blacksburg, VA, USA) were used for rapid testing for *Cryptosporidium* spp antigen (appendix 2 p 3). Lateral-flow strip testing was done by laboratory technicians other than those who did LED-AP testing and was done at the Jimma Medical Centre laboratory, twice per week.

As there is no commonly accepted reference-standard test for asymptomatic or symptomatic *Cryptosporidium* spp infections, we used a composite reference panel that included quantitative IFAT (qIFAT) for detection of *Cryptosporidium* spp oocysts, ELISA for detection of *Cryptosporidium* spp antigen, and qPCR for detection of *Cryptosporidium* spp DNA (appendix 2 p 3). Quantitative cutoff values for the association of *Cryptosporidium* spp detection with diarrhoea were defined as the qPCR and qIFAT quantity that maximally discriminated case or control status in the case-control substudy and were given as target DNA copy number (qPCR) or oocysts (qIFAT) per g of wet stool.

Positive microbiological composite reference standard (MRS) was defined as two or more positive reference tests and negative MRS as two or more negative reference tests. Positive clinical composite reference standard (CRS) was defined as two or more positive reference tests (and greater than the quantitative cutoff value for association with diarrhoea) and negative CRS as two or more negative reference tests (or less than the quantitative cutoff value for association with diarrhoea). *Cryptosporidium* spp infection was defined as a diarrhoea case or non-diarrhoea control with positive MRS. Cryptosporidiosis was defined as a diarrhoea case with positive CRS.

Operational data were also collected and these included test turnaround times, cost of reagents and laboratory expendables used for LED-AP and ICLF strip testing, standardised report forms from supervision visits to both enrolment sites, and reports on internal quality assessment at both sites.

### Statistical analysis

For the diagnostic accuracy sample-size calculations, we assumed that the main limiting factor would be sensitivity, as its precision would depend on the number of children with cryptosporidiosis that we recruited into the study. With a margin of error of 10%, a power of 90%, and an assumed minimum acceptable sensitivity of 70% for a cryptosporidiosis test to be cost-effective,^24^ we estimated that a minimum of 75 children with cryptosporidiosis should be enrolled to achieve the necessary precision in sensitivity. Double data entry was done with EpiData (version 3.1). All data analyses were done using R (version 3.5.2). 95% CIs were used to represent statistical precision and a p value of less than 0·05 was considered significant.

Quantitative cutoff values designed to maximise discrimination between cases and controls were established for *Cryptosporidium* spp qPCR and qIFAT by analysis of all PCR and IFAT-positive cases and controls in the substudy, using a receiver operating characteristic curve analysis with pathogen quantity in stool as the discriminating variable and case or control status as the dependent variable (appendix 2 p 7).

Difference in average *Cryptosporidium* spp quantity (DNA copies per g of stool for qPCR and oocysts per g for qIFAT) between case and control samples was assessed by comparing mean averages in an unpaired Welch two-sample *t* test; if distributions were positively skewed, we used log transformation before the *t* test to achieve normality.

95% CIs for test sensitivity, specificity, and positive and negative predictive values were calculated by the Wilson method,^25^ and for positive and negative likelihood ratios with formulae from Simel and colleagues.^26^ The strength of association between *Cryptosporidium* spp detection and diarrhoea was approximated by the odds ratio in the case-control substudy. Spearman's rank correlation was used to quantify the association between semiquantitative scoring of positive AP slides (ie, one to nine, ten to 50, or more than 50 oocysts per magnification field of view) with quantitative results obtained by qPCR and qIFAT.

### Role of the funding source

The funders of the study had no role in study design, data collection, data analysis, data interpretation, or writing of the report. The corresponding author had full access to all the data in the study and final responsibility for the decision to submit for publication.

## Results

**Figure 2:**
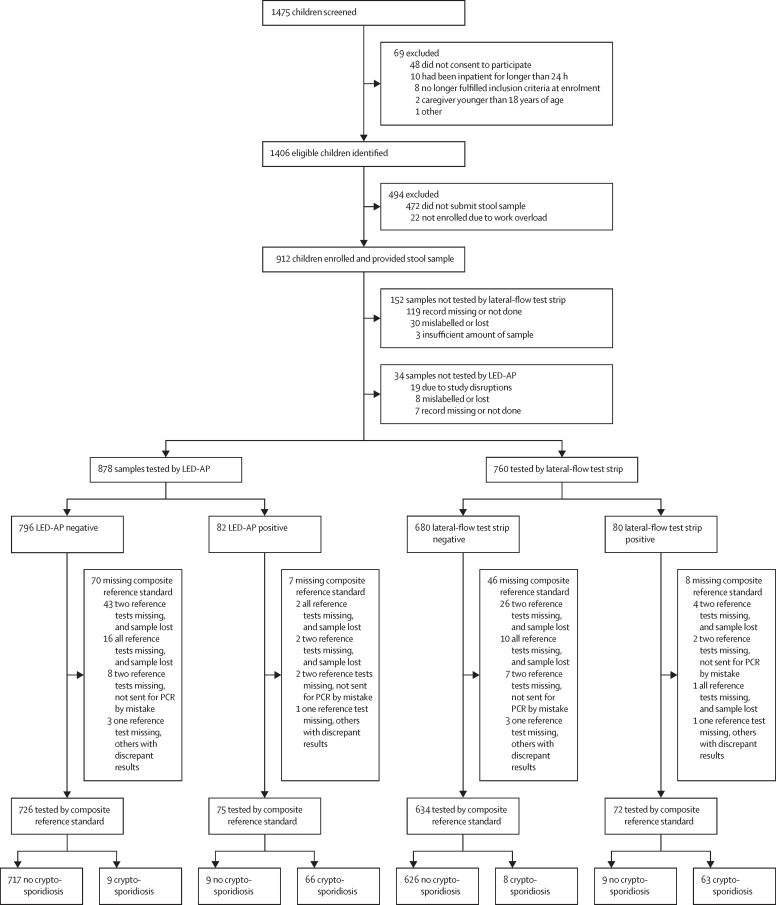
Study flowchart LED-AP=light-emitting diode fluorescence microscopy with auramine-phenol (AP) staining.

From Dec 22, 2016, to July 6, 2018, 1475 children with diarrhoea or dysentery were screened, 1384 were enrolled and, of these, 912 provided a stool sample (figure 2; table 1). All children who fulfilled the dysentery definition also fulfilled the diarrhoea definition. The case-control substudy^23^ enrolled 1134 children with diarrhoea (with 749 stool samples) and 946 controls (706 stool samples; appendix 2 pp 5–6).

**Table 1:** Demographic and clinical characteristics of children with diarrhoea in the diagnostic accuracy study

		**Participants**
Age in months, median (IQR)		12 (8–22)
Female sex		379/912 (42%)
Diarrhoea episode		
	Duration in days, median (IQR)	3 (2–5)*
	Acute episode (1–6 days)	745/907 (82%)
	Prolonged episode (7–13 days)	116/907 (13%)
	Persistent episode (14 days or longer)	46/907 (5%)
Moderate-to-severe diarrhoea		190/782 (24%)
Dysentery (visible blood in stool)		
	By caregiver report	78/910 (9%)
	By caregiver report or by laboratory technician inspection of stool†	92/910 (10%)
Severe acute malnutrition		86/911 (9%)
Stunted growth		200/907 (22%)
HIV positive		2/682 (<1%)
HIV exposed		2/682 (<1%)
Rotavirus vaccination		853/910 (94%)
Enrolment site Jimma Medical Centre		445/912 (49%)
Enrolment site Serbo Health Centre		467/912 (51%)

Data are n/N (%) unless otherwise stated. Denominators vary because of missing data for some participants (eg, not recorded or not answered).

* N=907.

† By gross inspection of the sample after it arrived in the clinical laboratory (data available from 637 samples).

*Cryptosporidium* spp were significantly associated with diarrhoea with all detection methods used, but the strength of the association varied by test modality (table 2). When comparing *Cryptosporidium* spp quantities in case and control samples from the substudy, the median PCR quantity was 4·7 × 10^5^ DNA copies per g of stool (IQR 0·7–36·8 × 10^5^) in case samples and 1·2 × 10^5^ DNA copies per g (0·2–10·2 × 10^5^) in control samples. The PCR quantity ratio of geometric means in case stools to control stools was 3·8 (95% CI 1·5–9·8). The ratio was similar for IFAT quantification (3·3; 95% CI 1·8–6·1; appendix 2 pp 8–9). After estimating qPCR and qIFAT quantitative cutoff values (appendix 2 p 7) we applied the clinical composite reference standard to classify cases as cryptosporidiosis or non-cryptosporidiosis for the diagnostic accuracy calculations. 77 (9%) of 878 samples tested with LED-AP and 54 (7%) of 760 samples tested with the lateral-flow test strip had incomplete or missing reference-standard results and were excluded from further analysis (figure 2).

**Table 2:** The association between various *Cryptosporidium* spp detection methods and diarrhoea in the case-control substudy

	**Positive test in cases of diarrhoea, n/N (%)**	**Positive test in controls, n/N (%)**	Odds ratio of positive test for diarrhoea (95% CI)*
**Index tests**			
LED-AP	66/717 (9%)	11/689 (2%)	6·25 (3·27–11·94)
Lateral-flow test strip	67/620 (11%)	13/563 (2%)	5·13 (2·80–9·39)
**Reference tests**			
ELISA	69/666 (10%)	12/680 (2%)	6·43 (3·45–11·99)
PCR (any detection)	104/652 (16%)	45/668 (7%)	2·64 (1·82–3·81)
qPCR (above cutoff)	67/652 (10%)	15/670 (2%)	5·00 (2·83–8·85)
IFAT (any detection)	72/681 (11%)	25/668 (4%)	3·04 (1·90–4·86)
qIFAT (above cutoff)	48/680 (7%)	7/668 (1%)	7·17 (3·22–15·97)
**Reference panels**			
MRS	66/669 (10%)	14/676 (2%)	5·18 (2·88–9·31)
CRS	62/669 (9%)	8/677 (1%)	8·54 (4·06–17·98)

Denominators vary because of missing data for some participants (eg, not recorded or not answered). LED-AP=light-emitting diode fluorescence microscopy with auramine-phenol (AP) staining. qPCR=quantitative PCR. IFAT=immunofluorescent antibody test. qIFAT=quantitative IFAT. MRS=microbiological composite reference standard. CRS=clinical composite reference standard.

* Wald intervals.

Of the 77 children with cryptosporidiosis, 25 (38%) of 66 children (clinical outcome data missing for 11) had moderate-to-severe diarrhoea (appendix 2 p 4) and of the 745 cases without cryptosporidiosis, 149 (23%) of 638 children (data missing for 107) had moderate-to-severe diarrhoea. On enrolment of children both with and without cryptosporidiosis, the median diarrhoea duration was 3 days (IQR 3–7 for children with cryptosporidiosis and 2–4 days for children without). Severe acute malnutrition (appendix 2 p 4) was diagnosed in nine (12%) of 77 children with cryptosporidiosis and 69 (9%) of 744 children without cryptosporidiosis.

In the primary analysis, LED-AP had a sensitivity for cryptosporidiosis of 88% (95% CI 79–94; 66 of 75 samples) and a specificity of 99% (98–99; 717 of 726 samples). The lateral-flow test strip had a sensitivity of 89% (79–94; 63 of 71 samples) and a specificity of 99% (97–99; 626 of 635 samples; table 3).

**Table 3:** Diagnostic accuracy of LED-AP and lateral-flow test strip for cryptosporidiosis

	**LED-AP**	**Lateral-flow test strip**
Cryptosporidiosis prevalence	9% (8–12)	10% (8–13)
Sensitivity	88% (79–94)	89% (79–94)
Specificity	99% (98–99)	99% (97–99)
Positive predictive value	88% (79–94)	87·5% (78–93)
Negative predictive value	99% (98–99)	99% (98–99)
Likelihood ratio of a positive test	71·0 (36·9–136·3)	62·6 (32·6–120·4)
Likelihood ratio of a negative test	0·1 (0·1–0·2)	0·1 (0·1–0·2)

Data are point estimates with 95% CI. Cryptosporidiosis was defined as a case of diarrhoea with a positive clinical composite reference standard. Absolute numbers are shown in the appendix (p 10). LED-AP=light-emitting diode fluorescence microscopy with auramine-phenol (AP) staining.

An exploratory comparison of LED-AP and the lateral-flow test strip showed no significant differences in sensitivity, specificity, or predictive values between the two diagnostic tests (appendix 2 p 11). We observed a higher prevalence of cryptosporidiosis at the Jimma enrolment site than at the Serbo site, but adjusting the positive and negative predictive values for local prevalence had little effect on the estimates (appendix 2 p 11).

**Figure 3:**
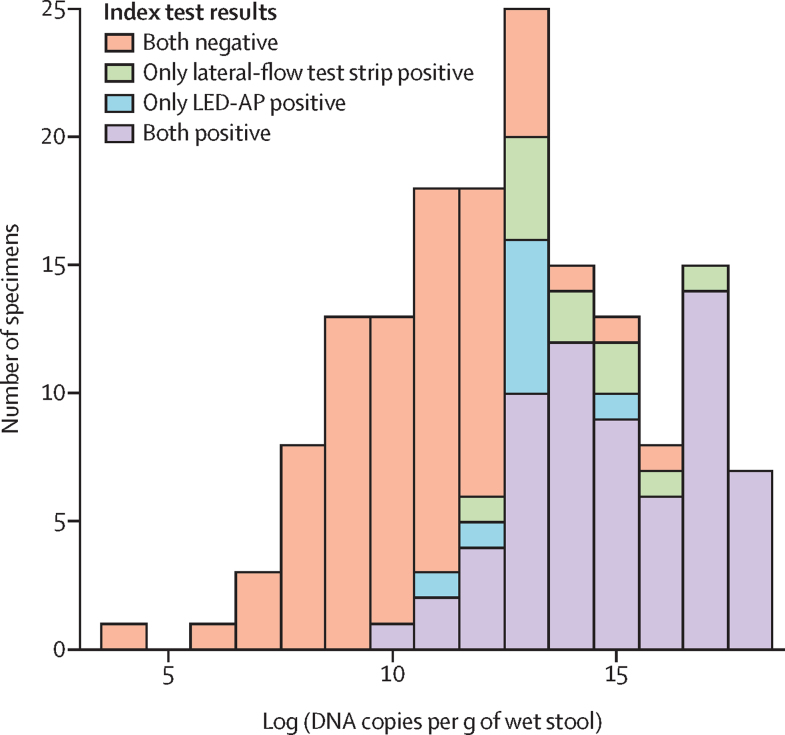
*Cryptosporidium* spp DNA quantity distribution Data from 127 diarrhoea cases and 37 diarrhoea-free controls; PCR quantity missing for two samples with positive microbiological composite reference standard. LED-AP=light-emitting diode fluorescence microscopy with auramine-phenol (AP) staining.

There was a positive correlation between semiquantitative grading of LED-AP slides (ie, one to nine, ten to 50, or more than 50 oocysts per magnification field of view) and quantitative results from qIFAT (Spearman's ρ 0·32, p=0·010) and qPCR (Spearman's ρ 0·27, p=0·011). The weakest IFAT-positive sample had only three oocysts in the IFAT sample well (approximately 75 oocysts per g of stool) and this sample was also positive by both LED-AP and the lateral-flow test strip. The lowest *Cryptosporidium* spp PCR quantity that was also positive by LED-AP was 13 836 DNA copies per g of stool; this was also the lowest PCR quantity that was positive by the lateral-flow test strip. Most PCR-positive samples that were negative by LED-AP and the test strip had low quantities of *Cryptosporidium* spp DNA (figure 3). Most false-negative index test results were in the lower range of positive by PCR, and there seemed to be a negative relationship between laboratory technician experience and false-negative test results (data not shown).

We did an exploratory analysis of the diagnostic accuracy of the index tests for asymptomatic infection by cross-tabulating LED-AP by the microbiological composite reference standard in controls with no diarrhoea. For asymptomatic infection, LED-AP had a sensitivity of 64% and a specificity of greater than 99%, and the lateral-flow test strip had a sensitivity of 79% and a specificity of greater than 99%, but these estimates had wide margins of uncertainty (appendix 2 p 11).

For turnaround time calculations, we used available data from LED-AP tests done on both case (n=878) and control (n=689) samples (n=1567; appendix 2 p 5). LED-AP was done on the day of stool collection in 1104 (77%) of 1431 tests, the following day in 309 tests (22%), and 2 or more days later in 18 tests (1%; data missing for 136). Total test turnaround time (ie, time from when the sample was obtained until the result was received) was available from 1322 (84%) of 1567 tests; of these, 607 results (46%) came back on the day the sample was obtained, 545 (41%) the following day, and the remaining 170 (13%) came back after 2 or more days. For results received the same day, test turnaround time ranged from 0·5 h to 7·8 h (median 3·5; IQR 1·7–4·9); a breakdown of turnaround times into subphases showed that median duration of preanalytical and postanalytical phases comprised the major fraction of total turnaround time (appendix 2 p 12).

Several operational issues relevant to implementation of LED-AP testing in routine care were identified during the study (appendix 2 p 13). The overall response of the clinical laboratory technicians after commencing AP staining for *Cryptosporidium* spp was encouraging, and they reported that operation and maintenance of the LED microscope was easier than or similar to that of conventional microscopes already in use. A specific benefit of the PrimoStar iLED microscope was the ease of switching between fluorescence and conventional light microscopy operation. A cost-per-test analysis, assuming expendables are purchased in bulk for approximately 500 tests, also factoring in labour time, estimated a cost of US$0·7 per LED-AP test, and $0·6 per lateral-flow strip test (excluding the cost of the test strip itself, as it is currently not yet available for purchase; appendix 2 pp 14–15).

## Discussion

We evaluated two tests suitable for near-patient use in children with community-acquired diarrhoea in a low-resource setting. In our combined population, children testing positive for *Cryptosporidium* spp by LED-AP or the lateral-flow test strip had 88% probability of having cryptosporidiosis, and those testing negative had 99% probability of not having cryptosporidiosis. Our study is the first adequately powered prospective diagnostic accuracy study for cryptosporidiosis in a consecutive series of children presenting to health-care facilities with diarrhoea in an LMIC. Testing was done by technicians who had received basic training and supervision, using the most widespread LED microscope in current use, and we found diagnostic accuracy consistent with estimates from a retrospective accuracy study in a high-income country.^11^ The *Cryptosporidium* EZ VUE lateral-flow test strips were easy to use and were as accurate as LED-AP, and have the advantage, by contrast with most ICLFs, that they do not require refrigeration.

We aimed for a representative sample by using broad inclusion criteria for all children aged younger than 5 years of age who presented with diarrhoea, by prospective enrolment irrespective of presenting complaint, and without excluding important and vulnerable subgroups, such as children with severe acute malnutrition or diarrhoea of prolonged duration. Test interpretation bias was addressed by full masking of technicians, and by applying the reference standard to all patients, irrespective of index test results.

Our study was insufficiently powered to estimate diagnostic accuracy for asymptomatic infection; these estimates therefore have a wide margin of uncertainty but indicate low sensitivity. Both tests failed to detect many only qPCR-positive samples from asymptomatic children, but we consider this finding as an advantage from a clinical point of view. Many low-level *Cryptosporidium* spp detections on qPCR in children with diarrhoea can probably be explained by diarrhoea from other infectious or non-infectious causes, combined with incidental detection of *Cryptosporidium* spp DNA. These detections could be explained by various reasons other than clinical infection: asymptomatic carriage, postinfectious shedding, or ingestion of oocysts that are no longer infectious or are from a *Cryptosporidium* species that does not infect humans. The main purpose of a test-and-treat strategy should be to detect the most likely causative agent of a diarrhoeal episode and thereby increase the chance of a useful clinical response. For the purpose of clinical drug trials for cryptosporidiosis, finding the right balance between sensitivity and specificity will increase the power to detect a clinical effect.

A range of factors should be considered when interpreting our findings. First, the study was deliberately done in an area of low HIV prevalence; however, as cryptosporidiosis is more common and severe in patients with impaired cellular immunity, the accuracy or predictive values of the tests might not be representative of settings with high prevalence of untreated HIV disease in children. Similarly, pretest and post-test probabilities for cryptosporidiosis might differ in vulnerable subgroups of children (eg, with prolonged or persistent diarrhoea, acute malnutrition, or severe diarrhoea), but we did not examine this in our study.

Second, missing composite reference-standard results for both LED-AP and the lateral-flow strip tests were somewhat higher than expected and, although the breakdown of reasons for missing tests (figure 2) indicate reasons unlikely to bias the accuracy estimates, missing-at-near-random is still an underlying assumption that warrants caution.

Third, we did not include tests for other diarrhoeal pathogens in this study because of cost constraints, which could lead to misclassification of some diarrhoeal episodes that were due to pathogens other than *Cryptosporidium* spp. Such misclassification could lead to bias and overestimation of specificity and positive predictive values. However, this bias would have a small effect, as there were few positive detections, and because previous reports indicate most high-quantity *Cryptosporidium* spp detections to be associated with diarrhoea, even when in mixed infections.^2^, ^27^ Detections of other acid-fast oocysts on LED-AP (eg, *Cystoisospora belli, Cyclospora cayetanensis*) occurred too infrequently to have any role (data not reported here).

Fourth, when estimating quantitative cutoff values for qIFAT and qPCR in the case-control substudy, we could not distinguish between new asymptomatic infections and shedding associated with a previous diarrhoeal episode. Although diarrhoea within the last month was reported for some children, the proportion was similar in cases and controls (appendix 2 p 6). Some epidemiological studies require a longer diarrhoea-free period for controls, but, in our opinion, the same criteria should apply to cases and controls. By using a short diarrhoea-free period (48 h), we increased the clinical validity of the quantitative cutoff values, and avoided excluding an important subgroup of cases from the study.

Fifth, the long test turnaround time for some samples could be a challenge for implementation but might, in part, be a study effect, because treatment was unavailable; ideally this should be re-evaluated as part of a test-and-treatment trial. Furthermore, test strip turnaround time was not measured, due to personnel constraints, but its hands-on time was similar to LED-AP. Considering the short hands-on time of LED-AP, the in-laboratory fraction of test turnaround time was large, but a much smaller contributor than the preanalytical and postanalytical phases. This breakdown of turnaround time is likely to reflect challenges in communication between laboratory and clinical personnel, and with sample transport. Preanalytical and postanalytical contributions to test turnaround time would need to be addressed before scaling up testing.

Lastly, as accuracies of specific ICLF kits vary considerably,^16^, ^17^, ^18^ we advise caution against extrapolating the accuracy estimates for the lateral-flow test strip to other ICLFs.

The minimum essential cost of any new cryptosporidiosis drug has been suggested as $2·0 per treatment course.^28^ The cost of diagnostics should be added to this, as empirical therapy would lead to unacceptably high rates of unnecessary treatment. In our setup, the added cost of testing amounted to about $0·7 per test for LED-AP, and considerably more for the rapid lateral-flow test strip when including the cost of the test strip itself. Although these cost estimates are well below the estimated cost for each cryptosporidiosis episode in LMICs,^29^ our results indicate that a comprehensive cost-effectiveness evaluation and an understanding of the local so-called diagnostic ecosystem would be required before large-scale rollout of either test.^30^

In conclusion, our results show that both LED-AP and the *Cryptosporidium* EZ VUE lateral-flow test strip have high diagnostic accuracy and operational features suitable for inclusion in test-and-treat interventions for cryptosporidiosis. Where LED microscopes are already available, LED-AP testing seems the more affordable, easy-to-implement, and sustainable option.

## Data sharing

Additional data (deidentified individual participant data) are available from the Norwegian Centre for Research Data, where requirements and conditions for access are also listed (under the Dokumentasjon link). Additional study documentation can be made available upon request to the corresponding author (ØHJ).

## Supplementary Materials

Amharic translation of the abstract
