# Supplementary material for: Performance and operational feasibility of two diagnostic tests for cryptosporidiosis in children (CRYPTO-POC): a clinical, prospective, diagnostic accuracy study
Source: Lancet Infect Dis. 2021 May;21(5):722–30. doi: 10.1016/S1473-3099(20)30556-9 (PMC8064915; doi:10.1016/S1473-3099(20)30556-9)
Supplement: Amharic translation of the abstract [file mmc1.pdf]

THE LANCET
Infectious Diseases

Supplementary appendix 1

This translation in Amharic was submitted by the authors and we reproduce it as supplied. It has not been peer reviewed. *The Lancet*'s editorial processes have only been applied to the original in English, which should serve as reference for this manuscript.

Supplement to: Johansen ØH, Abdissa A, Zangenberg M, et al. Performance and operational feasibility of two diagnostic tests for cryptosporidiosis in children (CRYPTO-POC): a clinical, prospective, diagnostic accuracy study. *Lancet Infect Dis* 2020; published online Dec 3. https://doi.org/10.1016/S1473-3099(20)30556-9.

በልጆች ላይ የሚከሰት ክሪፕቶስፖሪዲዮሲስ ህመም ለመመርመር የሚያስችሉ ሁለት የላቦራቶሪ መመርመሪያዎች የአፈፃፀም እና የአተገባበር አዋጭነት ጥናት፤

<u>በአጭሩ</u>

መግቢያ፤ ክሪፕቶስፖሪዲዮሲስ (Cryptosporidiosis) በለጋ ሕፃናት ላይ (ዕድሜያቸው ከ24 ወራት በታች በሆኑ) ተቅማጥን በማስከተል ከመታወቁም በላይ፤ የኑሮ ደረጃቸው ዝቅተኛ በሆኑ አገራት ይህንን ህመም የመመርመር አቅም በጣም ውስን ነው፡፡ይህንን ችግር ለመቅረፍ ለቲቢ ምርመራ እየዋለ ያለውን በብርሃን አሳላፊ ዲዮይድ ፍሎረሰንት ቀላሚ የኦራሚን-ፌኖል (Auramine-phenol) ማይክሮስኮፕ (LED-AP) በመገልገል ክሪፕቶስፖሪዲዮሲስንም መለየት ይቻላል። በቅርብ ጊዜም ማቀዝቀዣ ክምችት የማይፈለገው የላተራል-ፍሎው ምርመራ ለክሪፕቶስፖሪዲዮሲስ ልየታ ተዘጋጅቶ በአግልግሎት ላይ ውሏል። የዚህ ጥናታችንም አላማ በሕፃናት ላይ ተቅማጥ የሚያስከትለውን ክሪፕቶስፖሪዲዮሲስን ለመለየት የሚያስችሉትን የዲዮይድ ፍሎረሰንት ቀላሚ የኦራሚን-ፊኖል (Auramine-phenol) ማይክሮስኮፕ (LED-AP) እና የላተራል-ፍሎው የምርመራ (lateral-flow test) ዘዴዎችን በሽታውን በትክክለኛው የመመርመር ብቃት እና የአተገባበር አዋጭነትን መገምገም ነው ::

የጥናቱ መንገዶች፤ ይህ ጥናት የተካሄደው በኢትዮጵያ ውስጥ በጅማ እና አካባቢው ባሉ ሁለት የጤና አገልግሎት መስጫ ተቋማት ውስጥ የተቅማጥ በሽታ (ባለፉት 24 ሰዓታት ውስጥ ሶስት ወይም ከዚያ በላይ በከፊል የቀጠነ ዓይነምድር ናሙናዎች) ወይም ደም የተቀላቀበት ተቅማጥ (ባለፉት 24 ሰዓታት ውስጥ ቢያንስ አንድ በከፊል የቀጠነ እና ደም የተቀላቀለው የዓይነምድር ናሙና) ያለባቸውን፥ ዕድሜያቸው ከ 5 ዓመታት በታች የሆኑ ሕፃናት ላይ ክትትል በማድረግ ነው። የዓይነምድር ናሙናዎቹ የዲዮይድ ፍሎረሰንት ቀላሚ የኦራሚን-ፌኖል ማይክሮስኮፕ እና የላተራል-ፍሎው ምርመራ ተደርጎላቸዋል። የእያንዳንዱ ምርመራ ትክክለኙነት በተመጣጣኝ የማነፃፀሪያ መስፈርት (composite reference standard) መጠንን ባካተተ ኢሚውኖፍሎረሰንት ምርመራ (qIFAT) ፥ ኢላይዛ (ELISA) እና የፓራሳይት ዘረመል ቁጥር (qPCR) ገለልተኛ እና ስውር ንፅፅር ተገምቷል። ከተቅማጥ ጋር ተያይዞ ለሚመጣ ህመም የቁጥር ልኬት (cut off value) የተዘጋጀው በጅማ እና አካባቢው ካሉ 15 ወረዳዎች እና ከሰርቦ ዙሪያ ከሚገኙት 8 ወረዳዎች ከመጡ የክሪፕቶስፖሪዲዮሲስ ታማሚዎች ሲሆን ባለፉት 48 ሰዓታት ውስጥ ተቅማጥ የሌላቸውን እንደ ማነፃፀሪያ በመገልገል በየሳምንቱ መልካዓ ምድራዊ ክልልን ባገናዘበ የቤተሰብ፣ የእድሜ ክልል እና ሳምንታዊ የህክምና ምዝገባን መሰረት ያደረገ የማዛመድ ስራ ተከናውኖዋል።

የጥናቱ ውጤት፤ እኤአ ከዲሴምበር 22 ቀን 2016 እስከ ከጁላይ 6 ቀን 2018 ዓ.ም ባለው ግዜ ውስጥ ተቅማጥ ወይም ደም-የተቀላቀለበት ተቅማጥ ካለባቸው 912 ህጻናት እና ህመም ከሌለባቸው የማነፃፀሪያ ህፃናት ላይ ከተወሰደ 706 ናሙናዎች ላይ ምርመራ ተደርጓዋል፡፡ ለክሪፕቶስፖሪዲዮሲስ ህመም የተገመተው የማጣቀሻ መደበኛ የቁጥር ልኬቶች (reference-standard cutoff values) የነበሩት $2{\cdot}3\times10^{5}$ የዘረመል ቁጥር በአንድ ግራም እርጥብ ዓይነምድር በqPCR እና 725 oocysts በአንድ ግራም እርጥብ ዓይነምድር ለqIFAT ነበር። ለLED-AP ክሪፕቶስፖሪዲዮሲስ ህመም ያለባቸውን ህፃናት የመለየት አቅም 88% (95% CI 79-94፥ ከ75 ናሙናዎች ውስጥ 66) እና ህመሙ የሌላቸውን በትክክል የመለየት ሀቅም ደግሞ 99% (98 - 99፥ ከ726 ናሙናዎች ውስጥ 717)፤ የላተራል-ፍሎው ምርመራን በሚመለከት ደግሞ ህመሙ ያለባቸው በትክክል የመለየት አቅም 89% (79-94፥ ከ71 ናሙናዎች ውስጥ 63) እና ህመሙ የሌላቸውን በትክክል የመለየት አቅም ደግሞ 99% (97-99) ከ635 ናሙናዎች ውስጥ 626 የያዘ ነበር።

ትርጓሜ፤ የማይክሮስኮፕ **LED-AP** ምርመራ ህመምተኞችን በጥሩ ሁኔታ የመለየት አቅም ያለው በመሆኑ ዝቅተኛ ገቢ ባላቸው አካባቢዎች ውስጥ ከነባር የላብራቶሪ መሠረተ ልማቶች ጋር በቀላሉ በማቀናጀት ባለ ሁለትዮሽ ተግባር ቴክኖሎጂ ተደርጎ ሊወሰድ ይገባል። የላተራል-ፍሎው ምርመራም ተመሳሳይ አቅም ያለው ቢሆንም ማይክሮስኮፕ አያስፈልገውም፡፡ በእርግጥ የላተራል ፍሎው ምርመራ ገና በገበያው ላይ ስለማይገኝ ዋጋውም አይታወቅም ::

ወጪ፤ ይህንን ጥናት በገንዘብ የደገፉት የኖርዌይ ምርምር ካውንስል GLOBVAC ፈንድ፥ ቢል እና ሜሊንዳ ጌትስ ፋውንዴሽን፥ የኖርዌጂያን ሶሳይቲ ፎር ሜዲካል ማይክሮባዮሎጂ፥ የበርገን ዩኒቨርሲቲ እና ቬስትፎልድ ሆስፒታል ትረስት ናቸው፡፡
