# Supplementary material for: Performance and operational feasibility of two diagnostic tests for cryptosporidiosis in children (CRYPTO-POC): a clinical, prospective, diagnostic accuracy study
Source: Lancet Infect Dis. 2021 May;21(5):722–30. doi: 10.1016/S1473-3099(20)30556-9 (PMC8064915; doi:10.1016/S1473-3099(20)30556-9)
Supplement: Supplementary appendix 2 [file mmc2.pdf]

THE LANCET
Infectious Diseases

Supplementary appendix 2

This appendix formed part of the original submission and has been peer reviewed. We post it as supplied by the authors.

Supplement to: Johansen ØH, Abdissa A, Zangenberg M, et al. Performance and operational feasibility of two diagnostic tests for cryptosporidiosis in children (CRYPTO-POC): a clinical, prospective, diagnostic accuracy study. *Lancet Infect Dis* 2020; published online Dec 3. https://doi.org/10.1016/S1473-3099(20)30556-9.

Supplementary appendix for:

Performance and operational feasibility of two diagnostic tests for cryptosporidiosis in children (CRYPTO-POC): a clinical, prospective, diagnostic accuracy study

<u>Table of Contents</u>

HIV testing 2

Figure S1 and S2: *Cryptosporidium* LED-AP microscopy 2

Cryptosporidium EZ VUE antigen test strip 3

Cryptosporidium quantitative IFAT 3

C*ryptosporidium* ELISA 3

Cryptosporidium quantitative PCR 3

Definitions 4

Figure S3: Flow of participants and samples in the case-control substudy 5

Table S1: Demographic and clinical characteristics in the case-control substudy; diarrhoea cases and their frequency matched non-diarrhoea controls 6

Figure S4: Quantitative cutoff for qPCR – ROC curve 7

Figure S5: Quantitative cutoff for qIFAT – ROC curve 7

Figures S6 and S7: *Cryptosporidium* PCR quantity comparison in the case-control substudy – boxplot and histogram 8

Figures S8 and S9: *Cryptosporidium* IFAT quantity comparison in the case-control substudy – boxplot and histogram 9

Table S2: LED-AP results in relation to cryptosporidiosis; crosstables and accuracy calculations 10

Table S3: Test strip results in relation to cryptosporidiosis; crosstables and accuracy calculations 10

Table S4: Diagnostic accuracy of LED-AP versus test strip[1] for cryptosporidiosis (in reference to clinical composite reference standard) 11

Table S5: Prevalence-adjusted predictive values 11

Table S6: Diagnostic accuracy of LED-AP and *Cryptosporidium EZ-VUE* test strip for asymptomatic *Cryptosporidium* infection in children < 5 11

Table S7: Test turnaround times with breakdown in phases, for samples where results were received by study nurse the same day 12

Figure S10: Test turnaround times with breakdown in subphases 12

Table S8: Operational challenges with LED-AP *Cryptosporidium* staining identified during the study 13

Table S9: Cost per test calculation for LED-AP 14

Table S10: Cost per test calculation for antigen test strip 15

SOP: Auramine-phenol staining and light-emitting diode fluorescence microscopy (LED-AP) for *Cryptosporidium* 16

SOP: Preparation of reagents for auramine-phenol staining 18

Auramine-phenol positive and negative control sheet 20

Auramine-phenol internal quality control sheet 21

Checklist used for supervision visits to the LED-AP laboratory 22

Quality assurance and quality control of laboratory equipment 23

References 23

HIV testing

Guardians/caregivers of all participants were requested for permission for their children to have recommended HIV testing. Upon consent; first-line testing with the First Response HIV 1-2-O Card™ test (Premier Medical Corporation Ltd, Daman, India); for children younger than 18 months, positive test results were confirmed by PCR; for children older than 18 months, positive results were confirmed by a second test, Uni-Gold HIV™ (Trinity Biotech Manufacturing Ltd, Co. Wicklow, Ireland). HIV counselling and testing were done by routine clinical staff or trained study nurses. Information to caregivers and HIV treatment to children were offered according to routine care procedures.

Figure S1 and S2: *Cryptosporidium* LED-AP microscopy

(Also see standard operating procedure, page 16)

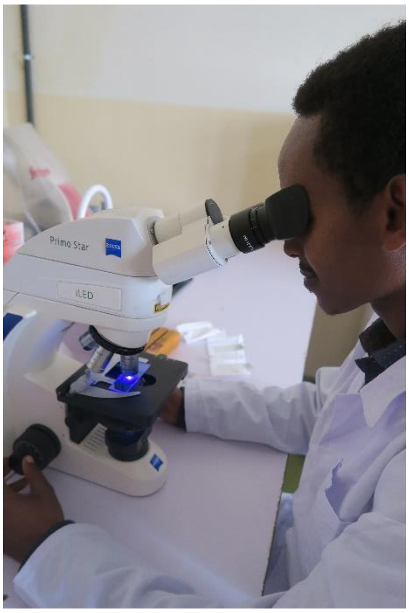

Figure S1: Examining auramine-phenol stained slides on the LED fluorescence microscope (ØHJ, personal photo)

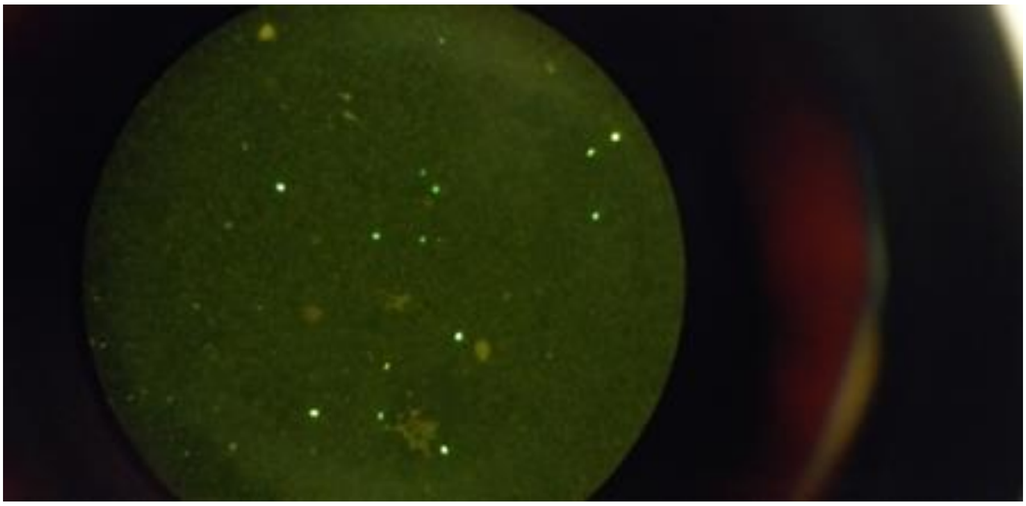

Figure S2: Brightly fluorescing circular/oval *Cryptosporidium* oocysts in an auramine-phenol stained stool smear, captured on site by smart phone camera held close to the LED microscope, through a 10x ocular and using a 40x objective; (YA, personal photo)

Cryptosporidium EZ VUE antigen test strip

Stool samples were mixed well by stirring with a wooden applicator stick, and about 50 µl (or about 0.05 g) faeces were transferred by pipette to a diluent tube, into which the test end of the lateral flow test strip was inserted, then inspected after 10 minutes. One operator read each test strip. The test was considered positive if both the test line and the control line were present, without any further confirmation or repeat testing, and as negative if only the control line was visible. Lack of a control line meant an invalid result and the procedure was repeated with a new test strip (once), by the same operator. However, invalid results were not seen during this study. Fleece and colleagues[1] have further details of the procedure, including a photo of a negative and positive test strip. Note that the *Cryptosporidium EZ VUE* test strip (TECHLAB®, Blacksburg, VA, USA) is currently not commercially available, and there is currently no plan to move it forward, although this could change in the future based on the perceived need (and market) for the product (Alice Houk-Miles, TechLab, Inc., personal communication).

Cryptosporidium quantitative IFAT

Immunofluorescent antibody test (IFAT) staining and microscopy were performed by a medical laboratory technician with special training in parasitology, in the JMC lab, twice weekly, on unconcentrated stool samples. Manually homogenized stool was transferred to one of three wells on a multispot microscope slide with a sterile metal wire loop calibrated to hold approximately 0.04g of stool. The samples were air-dried and then methanol fixed before staining with 5-15µl of monoclonal antibody for *Cryptosporidium* oocysts and *Giardia* cysts (Aqua-Glo G/C, Waterborne Inc., New Orleans, LA, USA) and then incubated in a humid chamber at room temperature (ranging from approx. 20 ºC to 30 ºC) for 40 minutes. The staining solution was then rinsed off with distilled water, one drop counterstain (Evans blue) added, and, after 1-minute incubation at room temperature, a drop of 1,4-diazabicyclo[2.2.2]octane anti-fade mounting medium (DABCO) and a coverslip was placed over the sample before microscopic examination. All *Cryptosporidium* oocysts in the sample were counted; if ≥ 100 oocysts were reached, the sample was diluted 1:10 in normal saline, re-stained and re-counted and this procedure was repeated until an oocyst count of <100 per well was registered. IFAT slides were kept at room temperature in a closed box and at monthly intervals the study investigator (ØHJ) selected 10 random slides for blinded review and assessed the quality of stains for the microscopy examinations; result discrepancies did not affect the recorded results of IFAT testing, but were used as the basis for feedback and on-site re-training for the lab technician.

C*ryptosporidium* ELISA

Enzyme-linked immunosorbent assay (ELISA) detection of *Cryptosporidium* antigen was performed in batches on frozen samples using a commercial assay (*Cryptosporidium II*, TECHLAB®, Blacksburg, VA, USA), with manual reading of the plates, and according to the manufacturer's protocol.

Cryptosporidium quantitative PCR

After completion of the field study, sample aliquots were shipped at -80°C from Ethiopia to Norway for TNA extraction and quantitative PCR.

Pre-treatment:

After thawing, stool samples were homogenized using a lab vortex mixer. An aliquot of stool was transferred to a buffer tube that was weighed before and after transfer to calculate the exact stool mass from which total nucleic acids were subsequently extracted, thereby allowing calculation of the number of DNA target copies detected per gram of stool. After thawing, stool samples were added to pre-made 500 µl S.T.A.R. buffer (Roche) + 500 µl BLB (MagNA Pure bacterial lysis buffer, Roche), and vortexed. The stool-buffer suspension was kept in an ultrafreezer until extraction.

Extraction:

The frozen stool-buffer suspension was heated for 15 minutes at 100°C, and centrifuged for 3 minutes at 13000g, before extraction. Nucleic acids were extracted from 500 µl of the pre-treated stool-buffer suspension, with MagNA Pure 96 instrument, MagNA Pure 96 DNA, and Viral NA Large Volume Kit, and eluted in 100 µl. An internal control (DNA process control kit, Roche) was added to every sample prior to extraction. Samples yielding a negative internal-control PCR result were diluted 1:4 in stool transport and recovery buffer (S.T.A.R. buffer, Roche) and bacteria lysis buffer (BLB, Roche), and then extracted again.

Real-time quantitative PCR:

The assay target gene was *Cryptosporidium* oocyst wall protein (COWP), using forward primer CAAATTGATACCGTTTGTCCTTCTG, reverse primer GGCATGTCGATTCTAATTCAGCT and probe FAM-TGCCA(T)ACAT(T)GT(T)GTCC-BBQ, as previously described by Van Lint et al.[2] Quantitative real-time PCR was run on a Light Cycler 480II, Roche, including primers and probes for the internal control (DNA process control kit, Roche). Standard curves were established by running qPCR on tenfold dilutions of Quantitative Genomic DNA from *Cryptosporidium parvum* (ATCC® PRA-67DQ™). Quantification cycles (Cq) are the PCR cycle values at which fluorescence from amplification exceeds the background, which acts as an inverse metric of quantity of nucleic acid. All detections with a Cq greater than 38 were deemed negative.

Conversion of Cq values to copy numbers:

All positive samples with Cq below Ct 38 were considered positive. The Cq were plotted against the external standard curve, allowing calculation of target copies/μl. A positive *Cryptosporidium parvum* control of known quantity was used as a calibrator for each run, to adjust for PCR differences.

Quality assurance:

We used good laboratory practice, automated pipetting of samples (primary sample handling, PSH) and DNA to PCR setup (MagNA Pure 96) to limit the possibility of laboratory contamination. Valid results required that positive, negative, and internal controls produced the expected results.

Definitions

- **Diarrhoea**: the passage of three or more watery or loose stools (looser than normal stools) within the preceding 24 h; the presence and duration of diarrhoea were assessed by caregiver recall.
- **Dysentery:** at least one loose stool with visible blood (or stains of blood) in the previous 24 h.
- **Severe acute malnutrition (SAM):** one or more of the following: weight-for-height z-score (WHZ) ≤ -3 of the WHO standard curves, mid-upper arm circumference (MUAC) ≤ 115 mm and presence of bilateral oedema involving at least the feet.
- **HIV status:** Human immunodeficiency virus infection status; based on HIV testing on enrolment or by previous testing as reported by the caregiver. Children below 18 months with an HIV-positive mother were considered HIV-exposed and uninfected if a PCR result for the child was negative or not available.
- **Stunting:** a length/height-for-age z-score ≤ -2 of the WHO standard curves.[3]
- **Moderate to severe diarrhoea:** diarrhoea together with very sunken eyes, abdominal skin pinch that returned very slowly (>2 s) or slowly (defined as ≤ 2s but abnormal), dysentery, received IV fluids or been admitted for any reason.[4]
- **Water/sanitation, assets and maternal education (WAM) index:** calculated as in the MAL-ED study; based on access to improved or unimproved water and/or sanitation, the presence or absence of eight household assets and maternal education.[5]
- **Rotavirus vaccination:** Rotavirus vaccine in Ethiopia is an oral vaccine (Rotarix™) that is given twice, usually at 6 weeks and 10 or 14 weeks of age. We defined the child as vaccinated against rotavirus if two doses had been received at least 4 weeks apart.
- **Sensitivity, specificity, positive and negative predictive values, likelihood ratio of a positive test, likelihood ratio of a negative test:** See table S2 and S3 (page 10, appendix)

Figure S3: Flow of participants and samples in the case-control substudy

(For details on screening and eligibility, see flow diagram in previous study.[6])

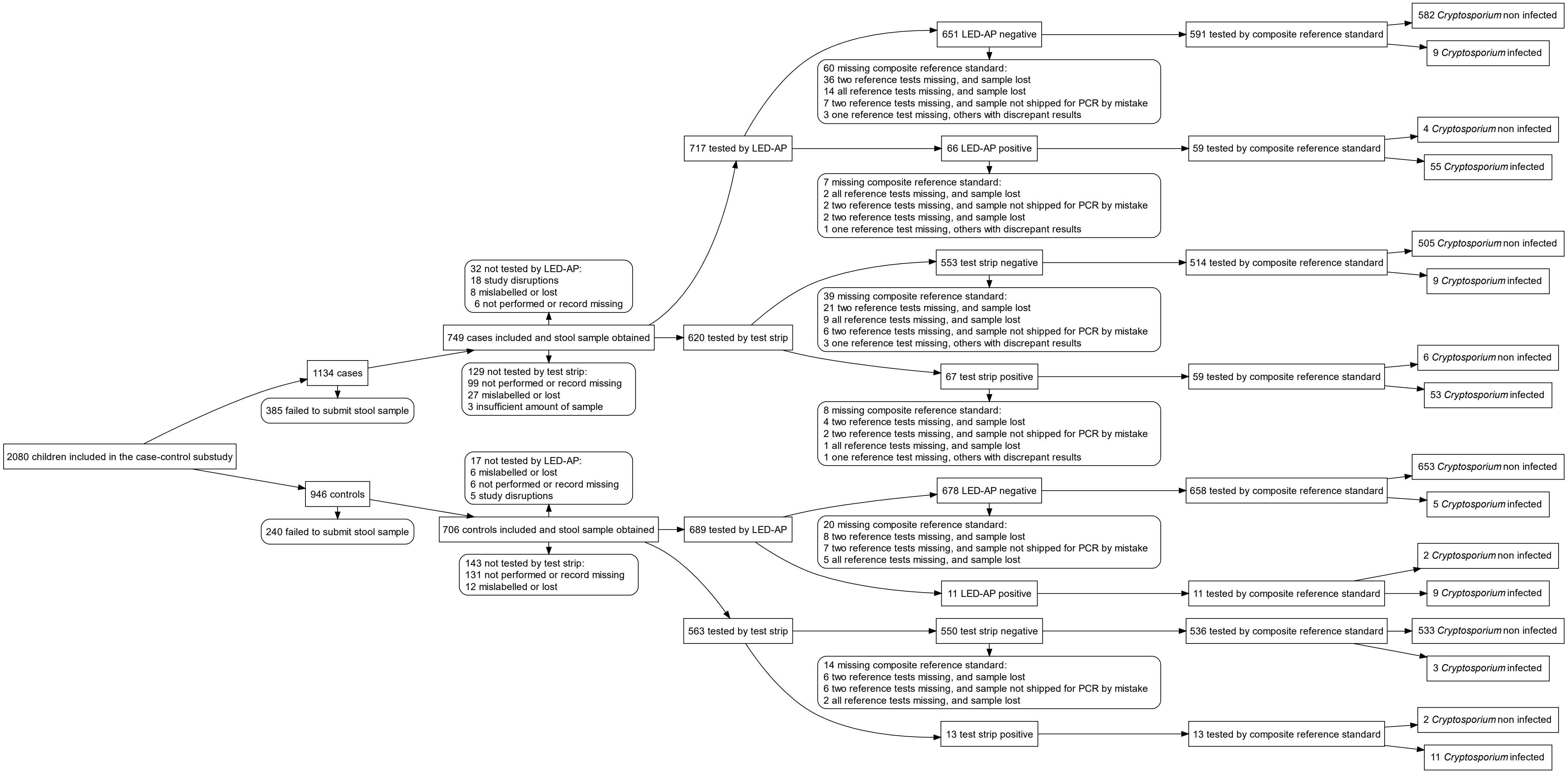

Table S1: Demographic and clinical characteristics in the case-control substudy; diarrhoea cases and their frequency matched non-diarrhoea controls

	**Diarrhoea cases** (n = 749)[1]	**Non-diarrhoea controls** (n = 706)[1]
Age, months; median (IQR)	13 (8-24)	16 (9-25)
Age, months; mean (SD)	17 (12)	19 (13)
Female gender; n (%)	308 (41%)	360 (49%)
Exclusive breastfeeding for < 6 months; n (%)	263/700 (38%)	242/658 (37%)
WAM[2] index < 0.50; n (%)	409/685 (60%)	366/728 (50%)
WAM index; mean (SD)	0.50 (0.20); N=728	0.44 (0.15); N=685
Health facility visit in previous month; n (%)	277/748 (37%)	177/706 (25%)
Diarrhoea episode in previous month; n (%)	123/744 (17%)	111/705 (16%)
Admitted in health facility since birth; n (%)	70/746 (9.4%)	61/706 (9.6%)
Previous treatment for malnutrition; n (%)	11/746 (1.5%)	8/705 (1.1%)
MUAC[3] ≤ 125 mm (children >6 months); n (%)	68/639 (11%)	13/629 (2.1%)
Severe acute malnutrition	50/748 (7%)	8/748 (1%)
HIV[4] positive	0/554 (0%)	0/264 (0%)
HIV exposed	1/554 (0.2%)	1/264 (0.4%)
Rotavirus vaccination	706/748 (94%)	671/702 (96%)

[1]Data are n/N (%), unless otherwise specified; denominators vary slightly because of missing data for some of the participants (e.g. not recorded, or not answered). [2]Water/sanitation, assets and maternal education (definitions, appendix). [3]Mid-upper arm circumference. [4]Human immunodeficiency virus.

Figure S4: Quantitative cutoff for qPCR – ROC curve

The cutoff that maximally discriminated case/control status was obtained by finding the threshold value (number in red, on the figure) for which the point on the ROC curve has the minimum distance (indicated by the red line in the figure) to the upper left corner (where sensitivity=1 and specificity=1). By Pythagoras' theorem, this distance is the square root of $(1\text{-sensitivity})^2+(1\text{-specificity})^2$.

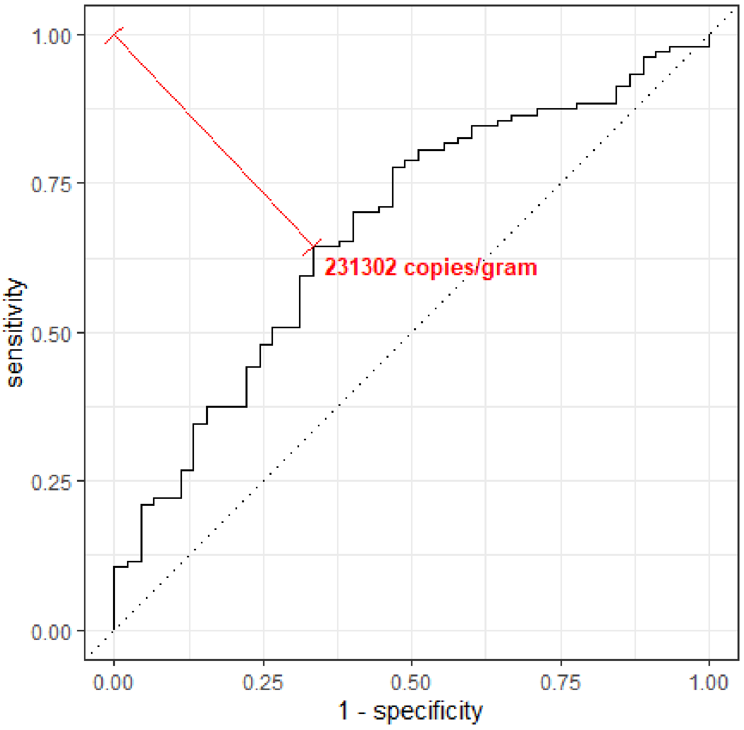

Figure S5: Quantitative cutoff for qIFAT – ROC curve

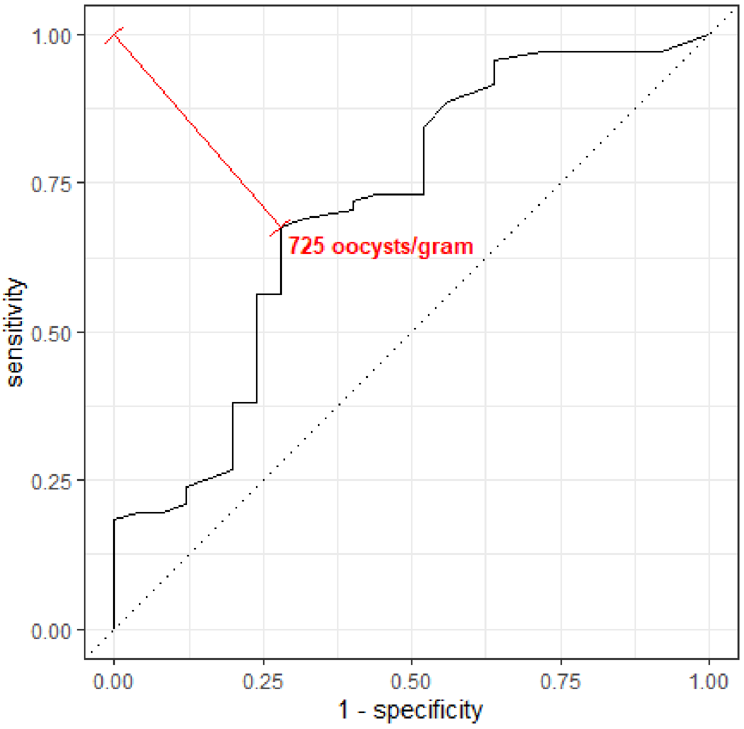

Figures S6 and S7: *Cryptosporidium* PCR quantity comparison in the case-control substudy – boxplot and histogram

The PCR quantity ratio in case to control stools was 3·8 (ratio of geometric means; 95% CI 1·5 to 9·8).

Horizontal and vertical dashed lines indicate the qPCR cutoff that maximally discriminates case/control status.

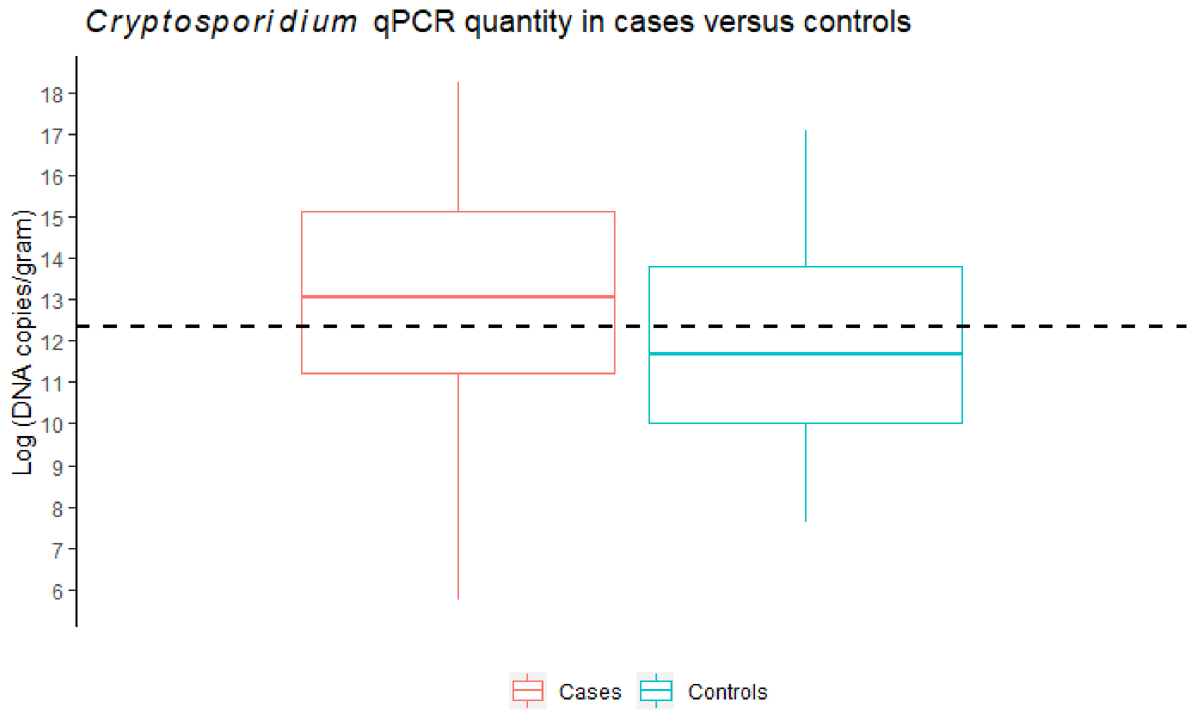

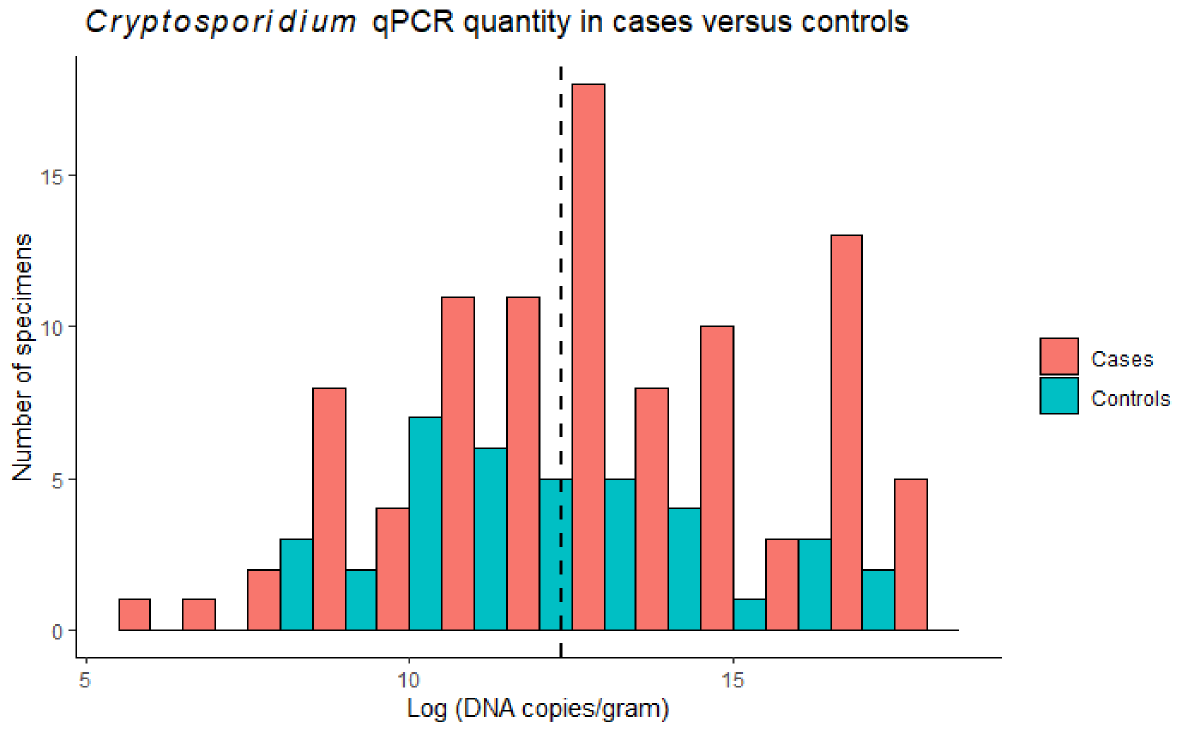

Figures S8 and S9: *Cryptosporidium* IFAT quantity comparison in the case-control substudy – boxplot and histogram

The IFAT quantity ratio in case to control stools was 3·3 (ratio of geometric means; 95% CI 1·8 to 6·1)

Horizontal and vertical dashed lines indicate the qIFAT cutoff that maximally discriminates case/control status.

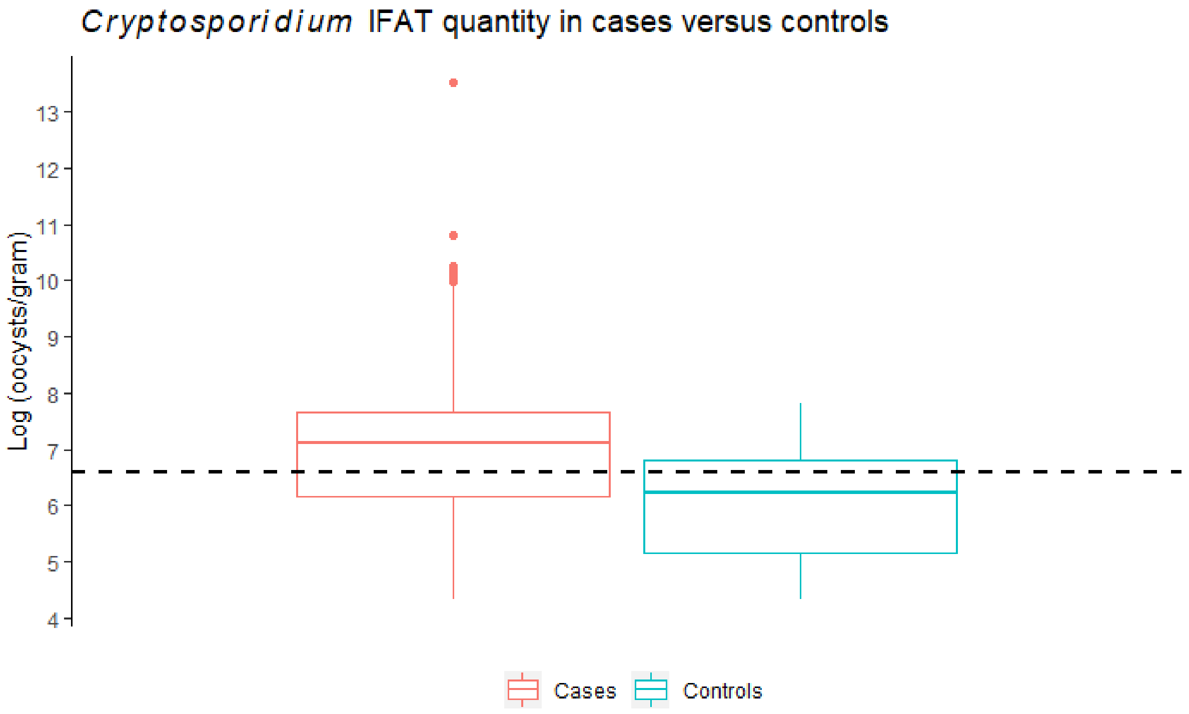

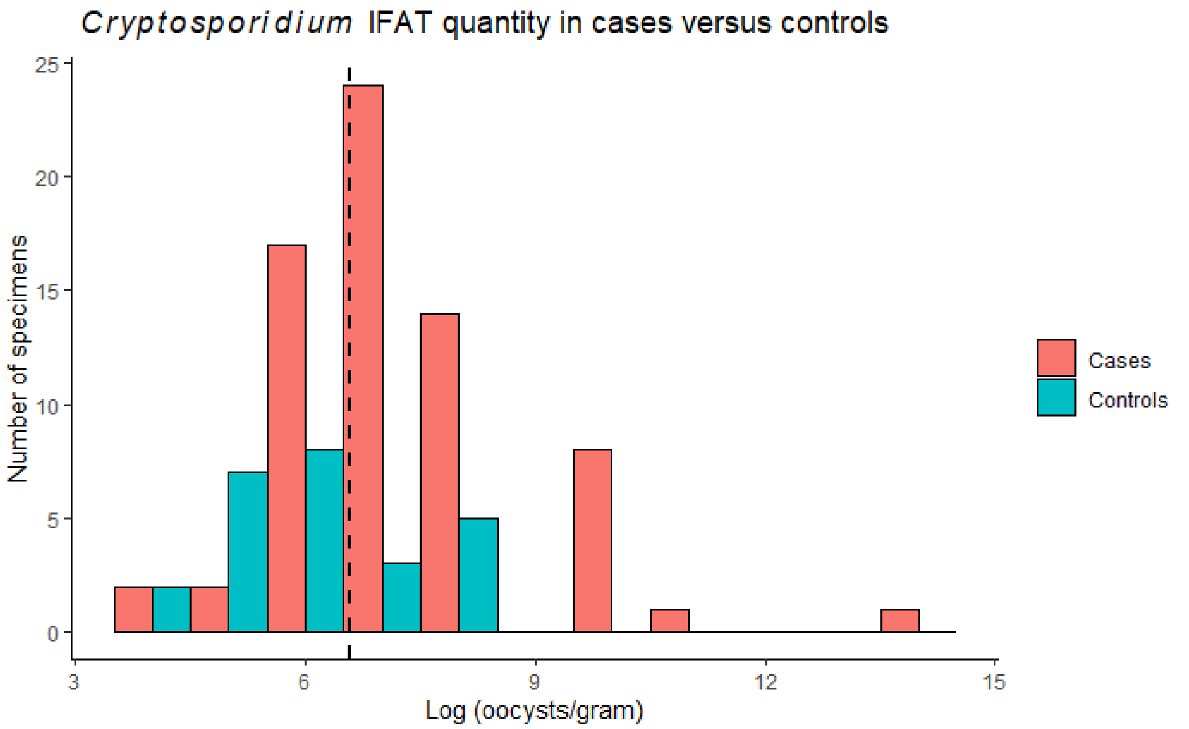

Table S2: LED-AP results in relation to cryptosporidiosis; crosstables and accuracy calculations

Cryptosporidiosis defined as a diarrhoea case with a positive clinical composite reference standard (CRS) test.

	Cryptosporidiosis		
LED-AP	Positive	Negative	Total
Positive	66 (tp)	9 (fp)	75
Negative	9 (fn)	717 (tn)	726
Total	75	726	801

The estimates in Table 3 in the main text were obtained using the following formulas:
Sensitivity = tp / (tp + fn) = 66 / 75 = 0.880
Specificity = tn / (fp + tn) = 717 / 726 = 0.988
Prevalence = (tp + fn) / (tp + fp + tn + fn) = 75 / 801 = 0.094
Positive predictive value = tp / (tp + fp) = 66 / 75 = 0.880
Negative predictive value = tn / (tn + fn) = 717 / 726 = 0.988
Likelihood ratio of a positive test = sensitivity / (1 – specificity) = 0.880 / (1 – 717 / 726) = 71.0
Likelihood ratio of a negative test = (1 – sensitivity) / specificity = (1 – 0.880) / (717 / 726) = 0.12

Table S3: Test strip results in relation to cryptosporidiosis; crosstables and accuracy calculations

Cryptosporidiosis defined as a diarrhoea case with a positive clinical composite reference standard (CRS) test.

	Cryptosporidiosis		
Test strip	Positive	Negative	Total
Positive	63 (tp)	9 (fp)	72
Negative	8 (fn)	626 (tn)	634
Total	71	635	706

The estimates in Table 3 in the main text were obtained using the following formulas:
Sensitivity = tp / (tp + fn) = 63 / 71 = 0.887
Specificity = tn / (fp + tn) = 626 / 635 = 0.986
Prevalence = (tp + fn) / (tp + fp + tn + fn) = 71 / 706 = 0.101
Positive predictive value = tp / (tp + fp) = 63 / 72 = 0.875
Negative predictive value = tn / (tn + fn) = 626 / 634 = 0.987
Likelihood ratio of a positive test = sensitivity / (1 – specificity) = (63 / 71) / (1 – (626 / 635) = 62.6
Likelihood ratio of a negative test = (1 – sensitivity) / specificity = (1 – 63 / 71) / (626 / 635) = 0.11

Table S4: Diagnostic accuracy of LED-AP versus test strip[1] for cryptosporidiosis (in reference to clinical composite reference standard)

	LED-AP	Test strip	P-value for difference	Difference
Sensitivity	90% (83 to 97)	88% (81 to 96)	1[2]	-1% (-10 to 7)
Specificity	99% (98 to 99)	99% (98 to 99)	1[2]	0% (-1 to 1)
Positive predictive value	87% (80 to 95)	87% (79 to 95)	0.96[3]	N/A
Negative predictive value	99% (99 to 100)	99% (98 to 100)	0.74[3]	N/A

[1]Note that estimates are slightly different to those in Table 3 in the main manuscript text, as this is a paired (i.e., head-to-head) analysis. Point estimates and 95% (Wald) confidence intervals[7], when applicable.
[2]Exact binominal test.[8]
[3]Generalized score statistic test.[9]

Table S5: Prevalence-adjusted predictive values

		Prevalence adjusted PPV		Prevalence adjusted NPV	
**Enrolment site**	**Prevalence[1]**	**LED-AP**	**Test strip**	**LED-AP**	**Test strip**
JMC	12.6% (9.7 to 16.2)	91.1% (82.5 to 95.7)	90.0% (80.9 to 95.1)	98.3% (97.0 to 99.0)	98.4% (97.1 to 99.1)
SHC	6.2% (4.3 to 9.0)	82.5% (72.4 to 89.4)	80.6% (69.9 to 88.1)	99.2% (98.2 to 99.6)	99.2% (98.2 to 99.7)

Point estimates and 95% confidence intervals. [1]Prevalence of cryptosporidiosis, defined as the proportion of diarrhoea cases with a positive CRS.

Table S6: Diagnostic accuracy of LED-AP and *Cryptosporidium EZ-VUE* test strip for asymptomatic *Cryptosporidium* infection in children < 5

	LED-AP	Test strip
Cryptosporidium infection prevalence	2.1% (1.3 to 3.5)	2.6% (1.5 to 4.2)
Sensitivity	64.3% (38.8 to 83.7)	78.6% (52.4 to 92.4)
Specificity	99.7% (98.9 to 99.9)	99.6% (98.6 to 99.9)
Positive predictive value	81.8% (52.3 to 94.9)	84.6% (57.8 to 95.7)
Negative predictive value	99.2% (98.2 to 99.7)	99.4% (98.4 to 99.8)
Likelihood ratio of a positive test	210.5 (50.0 to 886.7)	210.2 (51.3 to 861.0)
Likelihood ratio of a negative test	0.36 (0.18 to 0.72)	0.22 (0.08 to 0.59)

Point estimates and 95% confidence intervals. Asymptomatic *Cryptosporidium* infection defined as a non-diarrhoea control with a positive microbiological composite reference standard test (MRS).

Table S7: Test turnaround times with breakdown in phases, for samples where results were received by study nurse the same day

Turnaround times for samples reported the same day were subdivided into a pre-analytical phase (time from sample collection until arrival in the lab), analytical (time from sample arrival in lab until LED-AP completed) and post-analytical phase (time from LED-AP completion until result received by the study nurse).

	Both enrolment sites	JMC	SHC
Min; hrs	0.5	0.5	0.75
Max; hrs	7.8	7.8	7.3
Median; hrs (IQR)	3.5 (1.7 to 4.9)	1.8 (1.2 to 3.7)	4.6 (3.2 to 5.3)
Total TAT missing; n/N (%)	251/607 (41%)	240/415 (58%)	11/192 (6%)

Total turnaround time (TAT) by site, in hours

Figure S10: Test turnaround times with breakdown in subphases

Breakdown of turnaround time subphases, for samples analysed on the day of sample collection:

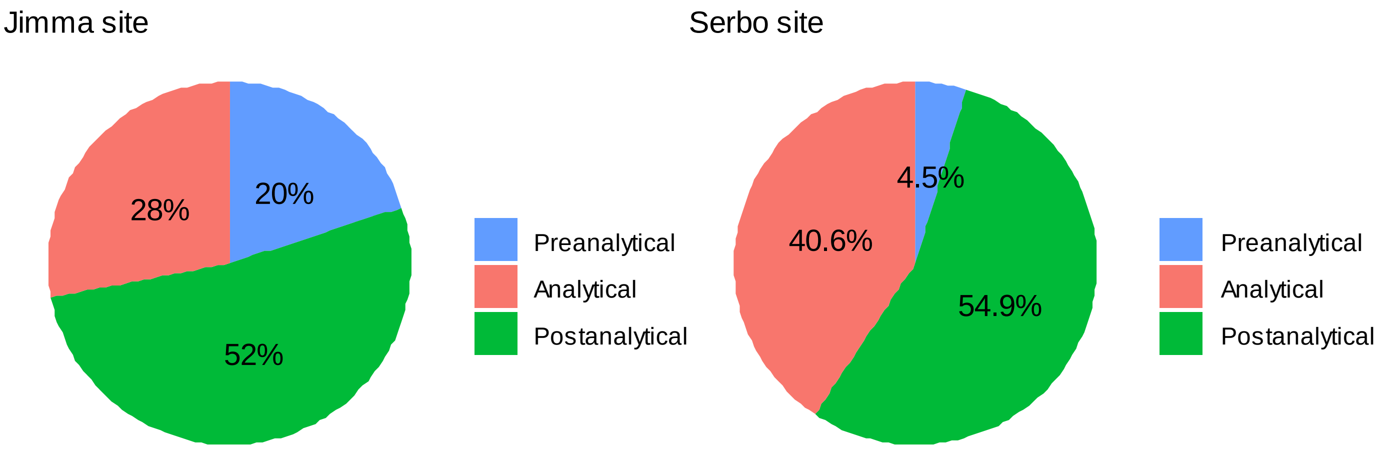

Table S8: Operational challenges with LED-AP *Cryptosporidium* staining identified during the study

Specific challenge	How the challenge was addressed during the study	Relevance to roll-out of LED-AP testing
**Preanalytical issues:**		
Failure to obtain stool sample because the patient left early	Information to the caretaker; transport compensation for returning with sample	Specific drug treatment, while waiting for sample provide ORS training and/or a separate waiting room for families
Long walking distance from the paediatric outpatient department and wards to the laboratory, and no clinical porter services	If the study nurse was busy, the lab tech was requested by phone to bring the sample to the lab (with ensuing delay)	Dedicated staff to transport samples, or set up small lab facility in the paediatric outpatients' department
**Analytical issues – general:**		
Uneven laboratory workload, with peaks just before lunch break and just before the end of the workday	Avoid sample backlog by immediate notification and transport	As above; ideally have both morning and evening shifts for lab staff
Power surge burned out the power unit in one of the LED microscopes	Voltage stabilizers installed (not routinely used in the clinical labs)	If not already in place, provide voltage stabilizers for all microscopes
Power interruptions in Serbo Health Centre as backup generator was broken and not fixed until 2 weeks later	Temporary analysis of samples in JUSTH lab instead	Solar panel and battery packs are available for most LED microscopes; general support for the lab facility (i.e., maintenance assistance)
Dwindling backup supply of AP staining reagent for several weeks; potassium permanganate difficult to import from abroad	Assistance from two regional clinical microbiology laboratories in different parts of the country	Cooperate on AP stain provision and contingency plans with the existing health infrastructure for tuberculosis diagnosis
Laboratory supervision interrupted for 2 weeks due to roadblocks	Phone communication with lab staff, supervision catch-up visit	Contingency plans for health service interruption; cell phone communication (and compensation for private use) with lab staff
Requests for follow-up training in the Serbo health centre lab	Extra training sessions organized in the JUSTH laboratory	Good support from centrally located labs to peripheral diagnostic labs
No darkroom for fluorescence microscopy	All LED microscopes equipped with plastic eye cups (eye strain low and high user satisfaction with these)	No need for darkroom, backup supply of plastic eye cups recommended
**Analytical issues – related to AP staining:**		
AP stain failing QC for 1 week in Serbo due to use of a plastic funnel that discoloured the stain	Staining reagent batch replaced; on-site supervision and training	Frequent and vigilant QC with blinded random review of slides recommended; on-site supervision and training recommended
Small 2-3µm circular or oval slightly fluorescent objects somewhat resembling *Cryptosporidium*	1. On-site training and SOPs in correct size measurement by micrometry; objects too small to (2-3µm vs 4-6 µm for *Cryptosporidium* spp.).<br>2. Specific stains for Microsporidia attempted (all negative)	Ideally equip all LED microscopes with insert eyepiece micrometer graticule, and provide SOPs and training in its use
Staining artefacts	1. Filtering of AP staining solutions, restaining of slides<br>2. If still staining artefacts, AP staining batch replaced<br>3. All AP staining bottles marked with expiry date	Written QC systems and designated QC officers for AP staining
Dust on microscope slides creating disturbing background fluorescence	1. Store all glass slides in closed box away from dust<br>2. Rinse slides with water before use<br>3. Do not use gauze or tissue paper to rinse off slides	Include similar points in SOP, provide appropriate paper for cleaning slides (i.e., lens paper or similar)
AP quantity grading discrepancies on blind review (+, ++, +++)	On-site feedback and training	Ideally include quantitative grading in blind review QC
AP slides haphazardly stored in drawers	More slide boxes provided	Ample supply of closed-lid plastic slide storage boxes for slide QC
Lab staff asking why internal oocyst contents give off fluorescence	Known phenomenon; as the AP stain likely is assumed to have nucleic acid staining properties in addition to acid-fast properties[10,11]	Laboratory bench aids with photographs of various types of appearance of *Cryptosporidium* oocysts in AP slides
**Postanalytical issues:**		
Nurse busy or otherwise unavailable to receive LED-AP result slip from the lab tech	Lab tech had to wait until study nurse was available as the patient's identity was unknown to the lab staff	In a non-research setting another member of the clinical staff could be approached to receive and act on the result
Nurse would sometimes call the lab tech and request result over phone	Temporary results given by phone, result form to follow	If possible, implement computer information systems to allow clinical staff to look up finished lab reports directly
The patient had already left when LED-AP result was available	Study nurse tried to reach the caretaker by phone, if possible	Possible "study effect"; if cryptosporidiosis treatment had been available, it would have been an added incentive to wait for the result

Table S9: Cost per test calculation for LED-AP

Cost item	Cost in Ethiopian Birr (ETB)	Unit	Quantity	Unit cost (ETB)	Total Cost (ETB)	Quantity used per test	Cost per test (ETB)	Average ETB to USD exchange rate during the study period	Cost per test (USD)	Notes
**Staining reagents**										
Auramine-O powder	55.35 for 100 mg	mg	100	0.5535	55.35	1.5	0.83	0.039763158	0.033	
Phenol crystals	147.08 for 1 kg	mg	1000	0.14708	147.08	1.5	0.22	0.039763158	0.009	(Could be slightly more expensive if < 1L purchased)
Potassium permanganate	65.0 for 100 mg	mg	100	0.65	65	1.5	0.98	0.039763158	0.039	
Methanol, absolute	240 for 1 L	ml	1000	0.24	240	2	0.48	0.039763158	0.019	(Could be slightly more expensive if < 1L purchased)
Ethanol, 96% (and HCl)	82.50 for 1 L	ml	1000	0.0825	82.5	0.5	0.04	0.039763158	0.002	(Could be slightly more expensive if < 1L purchased) Hydrochloric acid cost per test is 0.000 USD
**Other laboratory expendables**										
Glass microscope slides	38.50 for 50 pcs (1 pack)	pcs	50	0.77	38.5	1	0.77	0.039763158	0.031	
Applicator sticks	35.20 for 100 pcs (1 pack) =	pcs	100	0.352	35.2	1	0.35	0.039763158	0.014	
Sample collection cups	330 for 100 pcs	pcs	100	3.3	330	1	3.30	0.039763158	0.131	
Pasteur pipettes	300 for 500 pcs (1 carton)	pcs	500	0.6	300	1	0.60	0.039763158	0.024	
Gloves	118.90 for 100 (50 pairs)	pairs	50	2.378	118.9	1	2.38	0.039763158	0.095	
Tissue paper	210 for 1 big size roll	rolls	1	210	210	0.01	2.10	0.039763158	0.084	Assuming 1 roll is for 100 tests
**Hands-on labour time (**based on the approximate national average wage for nurses and lab technician working in the Ethiopian national health care system at the time; adjust numbers as needed.)										
Nurse (sample collection)	Weekly salary 900 ETB	min	6	0.375	2.25	1	2.25	0.039763158	0.089	
Lab tech (receipt and homogenization of sample)	Weekly salary 900 ETB	min	3	0.375	1.125	1	1.13	0.039763158	0.045	
Lab tech (staining procedure)	Weekly salary 900 ETB	min	6	0.375	2.25	0.5	1.13	0.039763158	0.045	This is the approximate hands-on time during the staining procedure, so not counting the waiting between manual steps. The "quantity used per test" is set to 0.5 as two slides would often be stained at the same time; adjust this number as needed.
Lab tech (microscopy)	Weekly salary 900 ETB	min	2	0.375	0.75	1	0.75	0.039763158	0.030	
Lab tech (reporting)	Weekly salary 900 ETB	min	4	0.375	1.5	1	1.50	0.039763158	0.060	
**Total**							**18.80**	0.039763158	**0.747**	

Table S10: Cost per test calculation for antigen test strip

Cost item	Cost in Ethiopian Birr (ETB)	Unit	Quantity	Unit cost (ETB)	Total cost (ETB)	Quantity used per test	Cost per test (ETB)	Average ETB to USD exchange rate during the study period	Cost per test (USD)	Notes
**Laboratory expendables**										
TechLab *Cryptosporidium EZ Vue* test strips	(see Notes)	1 test strip	1	25.15	25.14890801	1	25.15	0.039763158	1.000	As the test strip is not commercially available, this is a rough estimate (based on about 4 times the mean cost of malaria RDTs in health centres in Ethiopia[12]); adjust as needed
**Other laboratory expendables**										
Applicator sticks	35.20 for 100 pcs (1 pack)	pcs	100	0.352	35.2	1	0.35	0.039763158	0.014	
Sample collection cups	330 for 100 pcs	pcs	100	3.3	330	1	3.30	0.039763158	0.131	
Pasteur pipettes	300 for 500 pcs (1 carton)	pcs	500	0.6	300	1	0.60	0.039763158	0.024	
Gloves	118.90 for 100 (50 pairs)	pairs	50	2.378	118.9	1	2.38	0.039763158	0.095	
Tissue paper	210 for 1 big size roll	rolls	1	210	210	0.01	2.10	0.039763158	0.084	Assuming 1 roll is for 100 tests
**Hands-on labour time (**based on the approximate national average wage for nurses and lab technician working in the Ethiopian national health care system at the time; adjust numbers as needed.)										
Nurse (sample collection)	Weekly salary 900 ETB	min	6	0.375	2.25	1	2.25	0.039763158	0.089	
Lab tech (receipt and homogenization of sample)	Weekly salary 900 ETB	min	3	0.375	1.125	1	1.13	0.039763158	0.045	
Lab tech (test strip procedure)	Weekly salary 900 ETB	min	2	0.375	0.75	0.5	0.38	0.039763158	0.015	This is the approximate hands-on time, so not counting the waiting step. This assumes that an average of 2 samples will be received and processed at the same time
Lab tech (test strip interpretation)	Weekly salary 900 ETB	min	1	0.375	0.375	1	0.38	0.039763158	0.015	
Lab technician (reporting)	Weekly salary 900 ETB	min	4	0.375	1.5	1	1.50	0.039763158	0.060	
**Total**							**39.50**	0.039763158	**1.571**	

SOP: Auramine-phenol staining and light-emitting diode fluorescence microscopy (LED-AP) for *Cryptosporidium*

(Excerpt from the study Laboratory Manual; this standard operating procedure (SOP) was used for training of laboratory technicians in two half-day training sessions given one month apart; and as laboratory bench aids for the diagnostic laboratory)

Introduction
This fluorescent staining technique is used for the demonstration of *Cryptosporidium* oocysts, in faeces. Note that *Cyclospora cayetanensis* and *Cystoisospora belli* oocysts also stain by this method.

Safety considerations
Because of ocular and cutaneous toxicity, work with auramine-phenol should be performed using personal safety equipment (eye protection, gloves, clothing) in rooms with good ventilation.

Required equipment

- Microscope slides
- Methanol (absolute)
- Auramine-phenol solution (0.1% auramine-O) (ready-made commercial stain, or prepared according to the Ministry of Health protocol)
- 0.5% acid ethanol
- 0.5% potassium permanganate solution
- Pipettes
- Timer/stopwatch
- Distilled water or tap water (use a beaker, bottle with a plastic tube attached to it, or a plastic tube from the tap (gentle flow!)
- Positive control (*Cryptosporidium* oocysts, available from Waterborne Inc, mixed with faecal material)
- Negative control (faecal material known not to contain *Cryptosporidium* oocysts)

Method

a) Homogenize the stool sample as much as possible by stirring vigorously in the sample container with an applicator stick or loop
b) Prepare a smear and air dry, either in room temperature for 15-30 minutes, or in 37°C incubator (smears should be medium to thick)
c) Fix in absolute methanol for 1min, then air dry before proceeding with staining.
d) Flood the slide with Auramine-phenol solution (0.1% auramine-O) (ready-made commercial stain or prepared according to the MoH protocol) and leave the solution on the slide for 15 min. Do not heat.
e) Rinse with distilled water (or tap water if distilled water not available) from a beaker, or gently through a plastic tube attached to a bottle or a tap. Drain excess water from the slide.
f) Flood the slide with 0.5% acid ethanol and leave the destaining solution on the slide for 2min
g) Rinse with distilled water (or tap water if distilled water not available) from a beaker (not directly from the tap), or gently through a plastic tube attached to a bottle or a tap. Drain excess water from the slide.
h) Flood the slide with 0.5% potassium permanganate and leave the counterstain fluid on the slide for 2 minutes. The timing of this step is critical.
i) Rinse with distilled water (or tap water if distilled water not available) from a beaker (not directly from the tap), or gently through a plastic tube attached to a bottle or a tap. Drain excess water from the slide, and air dry. Do not blot because some blotting materials/paper may fluoresce.
j) Let the sample dry, either in room temperature (approximately 15-30 minutes) or in incubator at 37°C.
k) Examine with x 20 objective and x 10 eyepiece lens, and the PrimoStar iLED fluorescence microscope (blue light). The whole sample area should be examined for the presence of fluorescent oocysts. Suspicious objects can be re-examined with a 40x objective or with oil-immersion and the 100x objective.
l) Prepared slides should be kept in a closed slide box, at room temperature, for QC purposes.

Interpretation

Positive Result
Cryptosporidium oocysts (4-6 µm diameter) are ring or doughnut-shaped and fluoresce greeny-yellow (depending on the filter wavelengths) against a dark background. Putative oocysts may be measured by increasing the bright field light intensity and measuring the oocysts with a calibrated eye-piece graticule.

Note that *Cystoisospora belli* oocysts (about 32x16 µm, elongated oval body, tapered at both ends) and *Cyclospora cayetanensis* oocysts (8-10 m diameter, often variably stained) will also fluoresce using the auramine-phenol staining technique, due to the acid-fast properties of these organisms. Note that the oocysts of *Cystoisospora belli* and *Cyclospora cayetanensis* usually fluoresce less well than *Cryptosporidium* oocysts.

Oocyst counts (semi-quantitative method only):
Grade the samples after counting the average number of oocysts per 200x magnification field of view using the x20 objective (count the number of oocysts in minimum 10 fields, and divide by 10 to get the average number of oocysts per field)

Grade the samples after counting the number of oocysts per field of view using the x20 objective:

- 1-9 oocysts: +
- 10-50: ++
- >50: +++

Negative Result
No fluorescent objects with the correct shape or size for *Cryptosporidum*, *Cystoisospora belli* or *Cyclospora cayetanensis.*

Common interpretation challenges
Occasionally acid-fast objects in stool can fluoresce with AP stain, but they will not have the typical "doughnut" shape of *Cryptosporidium* spp. Yeast cells can fluoresce weakly but tend to lack the typical ring or doughnut shape of *Cryptosporidium*. In contrast to the large majority of fluorescent artefacts, the fluorescence is heterogeneously distributed in the interior of the oocysts. Refer to laboratory bench aids with photographs of the typical appearance of oocysts of *Cryptosporidium*, *Cystoisospora belli* and *Cyclospora cayetanensis*.

Quality control

Positive control
Cryptosporidium species oocysts; positive control material obtained from Waterborne Inc.

Negative control
A proven negative smear may be used as the negative control. Negative faecal control material can be obtained from Waterborne Inc.

Mix the positive and negative control vials before use.

Include a *Cryptosporidium* positive control and a negative control every time LED-AP microscopy is performed, minimum 1 positive and 1 negative slide per 10 slides that have been stained, or once per week, whichever comes first.

SOP: Preparation of reagents for auramine-phenol staining

The reagents used are the same as those recommended for auramine staining of sputum smears for acid-fast bacilli (e.g.,[13]).

(Excerpt from the study Laboratory manual standard operating procedure (SOP):)

Preparation of reagents – general

Batches of reagents should be prepared in adequate volumes according to need.

Equipment

- Balance, with a sensitivity of 0.1 g
- Brushes to clean bottles before reuse
- Containers for the newly prepared stains (dark amber glass bottles or plastic bottles)
- Distilled or purified water
- Flasks (conical or flat-bottomed), capacity at least 1 litre
- Filter papers, large (appropriate size for funnels)
- Funnels, large, for filling bottles
- Labels for bottles
- Stirring plate, heated, and magnetic stirrers
- Chemicals, see below

Auramine 0.1% - preparation

- Auramine 1.0 g (certified grade)
- Alcohol (denatured ethanol or methanol) 100.0 mL (technical grade)
- Phenol crystals 30.0g (analytical grade)
- Distilled water 870.0 ml

If liquefied phenol is to be used, adjust quantity as volume indicated by the manufacturer. First dissolve auramine in ethanol, then phenol crystals with water and mix both solutions. Mix only amounts that can be consumed within a few weeks, since the working solution is not stable in the long term, although the stock solution (1% auramine in alcohol) can be kept for longer (3 months). Thorough mixing for about one hour on a magnetic stirring plate is recommended, but the solution should not be heated.

Auramine stain – shelf life of working solution max 1 month

Label the bottle "0.1% auramine", add the date and sign with initials. The date the bottle is first opened must be written on the label.

Mix only amounts that can be consumed within a few weeks, since the working solution is not stable in the long term, although the stock solution (1% auramine in alcohol) can be kept for longer (3 months) "Stock and working solutions must be kept in dark bottles in the dark, and working solutions should be used within 1 month.

NB: If visible granules or precipitates form before 1 month has passed, the solution has expired, and you need to prepare a new working solution.

Auramine – safety

Wear gloves when handling patient specimens. Prepare slides from clinical specimens in safety cabinet. Use care when handling unstained slides to avoid touching infectious material.

Acid alcohol 0.5 % - preparation

- Hydrochloric acid 5 ml technical grade
- 70% ethanol 1000 ml

Use a 1-litre flask and slowly pour hydrochloric acid into alcohol.

Label the bottle "0.5% acid–alcohol", add the date and sign with initials. The date the bottle is first opened must be written on the label. This solution may be kept indefinitely.

Potassium permanganate 0.5% - preparation

- Potassium permanganate 5.0 g certified grade
- Distilled water 1000.0 ml

Label the bottle "0.5% potassium permanganate", add the date and sign with initials. The date the bottle is first opened must be written on the label. Solution should be used within 6 months.

Log-sheet for preparation of auramine, acid-alcohol, and counterstain:

**Auramine method**	**Quantity of reagent**	**Volume prepared**	**Date**	**Signature**
Auramine<br>Ethanol<br>Phenol<br>Distilled water<br>**Auramine 0.1%**	1.0 g<br>100 ml<br>30.0 g<br>1000 ml	1 litre		
Hydrochloric acid<br>Ethanol<br>**0.5% acid–alcohol**	5 ml<br>1000 ml	1 litre		
Potassium permanganate<br>Distilled water<br>**Counterstaining**	5 g<br>1000 ml	1 litre		

Auramine-phenol positive and negative control sheet

CRYPTO-POC study – Lab Manual Appendix – AURAMINE-PHENOL POSITIVE AND NEGATIVE CONTROL SHEET – v120417

AURAMINE-PHENOL POSITIVE AND NEGATIVE CONTROL SHEET – archive safely

NB: Mark all positive control slides with date and "AP POS CTR"; negative slides with date, and "AP NEG CTR"

Stain one *Cryptosporidium* positive and one *Cryptosporidium* negative control slide minimum 2 times per week

**Completed by (study staff number)** ___ ___ ___	**Completed by (full name)** _______________		**JUTH** ☐ **Serbo HC** ☐
**Date:** (DD/MM/YEAR): __ __ / __ __ / __ __ __ __	Positive control material ☐<br>Negative control material ☐	**Stain acceptable?** *(if no, take action!)*<br>Yes ☐ No ☐	**Comment/actions taken:**
**Date:** (DD/MM/YEAR): __ __ / __ __ / __ __ __ __	Positive control material ☐<br>Negative control material ☐	**Stain acceptable?** *(if no, take action!)*<br>Yes ☐ No ☐	**Comment/actions taken:**
**Date:** (DD/MM/YEAR): __ __ / __ __ / __ __ __ __	Positive control material ☐<br>Negative control material ☐	**Stain acceptable?** *(if no, take action!)*<br>Yes ☐ No ☐	**Comment/actions taken:**
**Date:** (DD/MM/YEAR): __ __ / __ __ / __ __ __ __	Positive control material ☐<br>Negative control material ☐	**Stain acceptable?** *(if no, take action!)*<br>Yes ☐ No ☐	**Comment/actions taken:**
**Date:** (DD/MM/YEAR): __ __ / __ __ / __ __ __ __	Positive control material ☐<br>Negative control material ☐	**Stain acceptable?** *(if no, take action!)*<br>Yes ☐ No ☐	**Comment/actions taken:**
**Date:** (DD/MM/YEAR): __ __ / __ __ / __ __ __ __	Positive control material ☐<br>Negative control material ☐	**Stain acceptable?** *(if no, take action!)*<br>Yes ☐ No ☐	**Comment/actions taken:**
**Date:** (DD/MM/YEAR): __ __ / __ __ / __ __ __ __	Positive control material ☐<br>Negative control material ☐	**Stain acceptable?** *(if no, take action!)*<br>Yes ☐ No ☐	**Comment/actions taken:**
**Date:** (DD/MM/YEAR): __ __ / __ __ / __ __ __ __	Positive control material ☐<br>Negative control material ☐	**Stain acceptable?** *(if no, take action!)*<br>Yes ☐ No ☐	**Comment/actions taken:**

Auramine-phenol internal quality control sheet

CRYPTO-POC study – Lab Manual Appendix – AP QC SHEET – v 050417

INTERNAL QC ON AP SLIDES	QC microscopy performed by (name):			Date:		
**ID number (on the slide)**	**Cryptosporidium oocysts**	**If Cryptosporidium oocysts are present, what is the oocyst count?**	**Cystoisospora belli**	**Cyclospora cayetanensis**	**Same result as on AP Lab Form?**	**Notes:**
_____-__	Present☐ Absent☐	+ ☐ ++ ☐ +++ ☐	Present☐ Absent☐	Present☐ Absent☐	Yes ☐ No ☐	
_____-__	Present☐ Absent☐	+ ☐ ++ ☐ +++ ☐	Present☐ Absent☐	Present☐ Absent☐	Yes ☐ No ☐	
_____-__	Present☐ Absent☐	+ ☐ ++ ☐ +++ ☐	Present☐ Absent☐	Present☐ Absent☐	Yes ☐ No ☐	
_____-__	Present☐ Absent☐	+ ☐ ++ ☐ +++ ☐	Present☐ Absent☐	Present☐ Absent☐	Yes ☐ No ☐	
_____-__	Present☐ Absent☐	+ ☐ ++ ☐ +++ ☐	Present☐ Absent☐	Present☐ Absent☐	Yes ☐ No ☐	
_____-__	Present☐ Absent☐	+ ☐ ++ ☐ +++ ☐	Present☐ Absent☐	Present☐ Absent☐	Yes ☐ No ☐	
_____-__	Present☐ Absent☐	+ ☐ ++ ☐ +++ ☐	Present☐ Absent☐	Present☐ Absent☐	Yes ☐ No ☐	
_____-__	Present☐ Absent☐	+ ☐ ++ ☐ +++ ☐	Present☐ Absent☐	Present☐ Absent☐	Yes ☐ No ☐	
_____-__	Present☐ Absent☐	+ ☐ ++ ☐ +++ ☐	Present☐ Absent☐	Present☐ Absent☐	Yes ☐ No ☐	
_____-__	Present☐ Absent☐	+ ☐ ++ ☐ +++ ☐	Present☐ Absent☐	Present☐ Absent☐	Yes ☐ No ☐	

Checklist used for supervision visits to the LED-AP laboratory

(excerpt of relevant sections from the study manuals:)

LABORATORY

- Documentation
    - Laboratory manual
    - Laminated LED-AP SOP bench aid on the wall
    - Laminated size measurement bench aid
    - Laminated stool microscopy bench aids
    - AP positive and negative control sheet
    - Result slip for LED AP
    - Check that LED-AP Result Sheets have been filled in correctly
- Check the supplies and the expiry dates (if relevant)
    - Stool collection tubes
    - Nappies, potties and plastic film for stool collection
    - Stains/reagents:
        - Auramine-phenol (working solution max shelf life 1 month!)
        - Acid alcohol
        - Potassium permanganate
        - Methanol
        - Normal saline for wet microscopy
    - Positive control (*Cryptosporidium* oocysts in suspension, for staining)
    - Negative stool control, for staining
    - Glass slides
    - Pipettes
    - Cover slips
    - Applicator sticks or plastic loops
    - Lens paper, lens cleaning solution
    - Oil for immersion microscopy
- Primostar iLED microscope checklist:
    - Plastic eye cups
    - Extra eyepiece with size measurement graticule
    - Lenses and the mechanical stage clean?
    - Is the protective plastic hood present and in use?

Quality assurance and quality control of laboratory equipment

(excerpt from the Study Lab Manual:)

QA and QC of Laboratory Equipment

Routine housekeeping should be in place and adhered to in order to monitor the status of equipment, machines, and their documents. A team consisting of the PIs, the laboratory technician, and microbiologist will frequently check the housekeeping documents, availability of manuals, and maintenance of equipment, machines and its relevant records considering the following issues as QC/QA.

1. Temperature chart for the fridges should be maintained. It should be recorded twice a day, always before opening the fridge door.
2. Laboratory User manuals should be read by the lab persons and discussed among them. The person who will handle the equipment should understand every point in the manuals.
3. Expiration dates of all reagents should be checked regularly, opening date of the reagents with initials should be labelled on the containers.
4. Reagents should be available and up to date. Orders should be placed well in advance to avoid shortages. Availability of supplies should be checked in-country and import delays, customs issues, should be anticipated during shipment.
5. Backup power should be available for the fridges.
6. Ensure that the voltage stabilizers are correctly installed.

References

1. Fleece ME, Heptinstall J, Khan SS, et al. Evaluation of a rapid lateral flow point-of-care test for detection of *Cryptosporidium*. *Am J Trop Med Hyg* 2016; **95**(4): 840-1.
2. Van Lint P, Rossen JW, Vermeiren S, et al. Detection of *Giardia lamblia*, *Cryptosporidium* spp. and *Entamoeba histolytica* in clinical stool samples by using multiplex real-time PCR after automated DNA isolation. *Acta Clin Belg* 2013; **68**(3): 188-92.
3. WHO Multicentre Growth Reference Study Group. WHO Child Growth Standards: Length/height-for-age, weight-for-age, weight-for-length, weight-for-height and body mass index-for-age: Methods and development. *Geneva: World Health Organization (312 pages)* 2006.
4. Kotloff KL, Blackwelder WC, Nasrin D, et al. The Global Enteric Multicenter Study (GEMS) of diarrheal disease in infants and young children in developing countries: epidemiologic and clinical methods of the case/control study. *Clin Infect Dis* 2012; **55 Suppl 4**: S232-45.
5. Psaki SR, Seidman JC, Miller M, et al. Measuring socioeconomic status in multicountry studies: results from the eight-country MAL-ED study. *Popul Health Metr* 2014; **12**(1): 8.
6. Zangenberg M, Johansen OH, Abdissa A, et al. Prolonged and persistent diarrhoea is not restricted to children with acute malnutrition: an observational study in Ethiopia. *Trop Med Int Health* 2019; **24**(9): 1088-97.
7. Wald A. Tests of statistical hypotheses concerning several parameters when the number of observations is large. *Transactions of the American Mathematical Society* 1943; **54**(3): 426-82.
8. Zhou X-H, Obuchowski NA, McClish DK. Statistical methods in diagnostic medicine. 2nd ed: John Wiley & Sons, Hoboken, New Jersey; 2011.
9. Leisenring W, Alonzo T, Pepe MS. Comparisons of predictive values of binary medical diagnostic tests for paired designs. *Biometrics* 2000; **56**(2): 345-51.
10. Hanscheid T, Cristino JM, Salgado MJ. Screening of auramine-stained smears of all fecal samples is a rapid and inexpensive way to increase the detection of coccidial infections. *Int J Infect Dis* 2008; **12**(1): 47-50.
11. Oster G. Fluorescence de l'auramine O en presence d'acide nucleique. *C R Hebd Seances Acad Sci* 1951; **232**(18): 1708-10.
12. Hailu A, Lindtjørn B, Deressa W, Gari T, Loha E, Robberstad B. Economic burden of malaria and predictors of cost variability to rural households in south-central Ethiopia. *PLoS One* 2017; **12**(10): e0185315.
13. Tuberculosis Control Assistance Project (TBCAP). Standard operating procedures (SOPs) - Module 27 - Auramine staining. http://www.stoptb.org/wg/gli/sops.asp (accessed 8 Feb 2020).
